# Identifying the Involvement of Pro-Inflammatory Signal in Hippocampal Gene Expression Changes after Experimental Ischemia: Transcriptome-Wide Analysis

**DOI:** 10.3390/biomedicines9121840

**Published:** 2021-12-05

**Authors:** Galina T. Shishkina, Natalia V. Gulyaeva, Dmitriy A. Lanshakov, Tatyana S. Kalinina, Mikhail V. Onufriev, Yulia V. Moiseeva, Ekaterina V. Sukhareva, Vladimir N. Babenko, Nikolay N. Dygalo

**Affiliations:** 1 Laboratory of Functional Neurogenomics, Federal Research Center Institute of Cytology and Genetics, Siberian Branch of the Russian Academy of Science, 630090 Novosibirsk, Russia; lanshakov@bionet.nsc.ru (D.A.L.); kalin@bionet.nsc.ru (T.S.K.); sev@bionet.nsc.ru (E.V.S.); bob@bionet.nsc.ru (V.N.B.); dygalo@bionet.nsc.ru (N.N.D.); 2 Laboratory of Functional Biochemistry of the Nervous System, Institute of Higher Nervous Activity and Neurophysiology, Russian Academy of Sciences, 117485 Moscow, Russia; nata_gul@ihna.ru (N.V.G.); mikeonuf1@rambler.ru (M.V.O.); jumois@ihna.ru (Y.V.M.); 3 Research and Clinical Center for Neuropsychiatry of Moscow Healthcare Department, 115419 Moscow, Russia

**Keywords:** RNA-seq, differentially expressed genes, middle cerebral artery occlusion, lipopolysaccharide, hippocampus

## Abstract

Acute cerebral ischemia induces distant inflammation in the hippocampus; however, molecular mechanisms of this phenomenon remain obscure. Here, hippocampal gene expression profiles were compared in two experimental paradigms in rats: middle cerebral artery occlusion (MCAO) and intracerebral administration of lipopolysaccharide (LPS). The main finding is that 10 genes (*Clec5a, CD14, Fgr, Hck, Anxa1, Lgals3, Irf1, Lbp, Ptx3, Serping1*) may represent key molecular links underlying acute activation of immune cells in the hippocampus in response to experimental ischemia. Functional annotation clustering revealed that these genes built the same clusters related to innate immunity/immunity/innate immune response in all MCAO differentially expressed genes and responded to the direct pro-inflammatory stimulus group. The gene ontology enrichment and Kyoto Encyclopedia of Genes and Genomes pathway analyses also indicate that LPS-responding genes were the most abundant among the genes related to “positive regulation of tumor necrosis factor biosynthetic process”, “cell adhesion”, “TNF signaling pathway”, and “phagosome” as compared with non-responding ones. In contrast, positive and negative “regulation of cell proliferation” and “HIF-1 signaling pathway” mostly enriched with genes that did not respond to LPS. These results contribute to understanding genomic mechanisms of the impact of immune/inflammatory activation on expression of hippocampal genes after focal brain ischemia.

## 1. Introduction

Acute immune/inflammatory activation in response to cerebral ischemic stroke has been suggested to play a dual role. Primarily, neuroinflammation is essential for removing of dead cells from the damaged areas and inducing brain repair mechanisms [1]. However, excessive neuroinflammation is believed to become a key factor contributing to further brain injury. Enhancement of neuroinflammation by injections of a lipopolysaccharide (LPS), bacterial cell wall endotoxin, augmented ischemic brain damage area [2,3,4]. Notably, after the onset of the first symptoms of stroke, an increase in the level of endotoxin was reported, presumably formed from Gram-negative bacteria of the respiratory, gastrointestinal, and urinary tracts [5].

Using middle cerebral artery occlusion (MCAO), a widely used model of ischemia in rodents, Uchida et al. observed neurodegenerative consequences also in the hippocampus, a brain region not directly affected by ischemia but demonstrating a remote damage [6]. The delayed cell death in the hippocampus is implicated in the development of post-stroke psychopathology, including cognitive impairment [7]. Remote hippocampal damage, most probably mediated by neuroinflammation, appears to be a common mechanism of different focal brain injuries, with stroke and brain trauma being most studied situations [8,9]. However, precise mechanisms of pro-inflammatory stroke effects on the hippocampus remain obscure. 

Identification of changes in post-stroke gene expression patterns and their time course in the hippocampus is essential for understanding molecular pathways involved in the development of stroke-induced delayed cognitive and affective disturbances. Cerebral ischemia is accompanied with global changes in expression of numerous genes not only in the cortical ischemic and surrounding areas [10], but also in the hippocampus [11,12]. Among these genes, inflammatory- and apoptosis-related genes were revealed. Central injections of LPS also resulted in hippocampal gene expression changes [13].

In the present study, we compared hippocampal gene expression profiles in two experimental paradigms in rats: focal brain ischemia (MCAO) and intracerebral LPS administration. The aim was to get insight into the involvement of stroke-induced inflammatory activation in remote effects of stroke on the hippocampus by determining genes directly affected by pro-inflammatory stimuli. Since MCAO causes severe neuronal damage in the ipsilateral striatum in rats [14], we have chosen striatum for central LPS administration according to the published protocol for acute rat model of local neuroinflammation in this structure [15]. Bioinformatics analyses, including gene ontology (GO), KEGG (Kyoto Encyclopedia of Genes and Genomes) pathway, and functional clustering analysis, were performed for differentially expressed genes (DEGs). Three lists of DEGs used for these analyses included: (a) all DEGs; (b) DEGs that responded to both MCAO and LPS; (c) DEGs that responded to MCAO, but not to LPS. 

## 2. Materials and Methods

### 2.1. Animals

Male Wistar rats (2.5 months of age) were used in the experiments. Animals were housed individually in polycarbonate cages (27.7 × 44 × 15 cm = w × l × h) with free access to food and water.

All animal use procedures were supervised and specifically approved by the ethics committee of the Institute of Cytology and Genetics in accordance with the guidelines of the Ministry of Public Health of Russia (supplement to order N 267 of 19 June 2003) and the European Council Directive (86/609/EEC). The middle cerebral artery occlusion and LPS administration into striatum included all measures to minimize rat suffering. 

### 2.2. Middle Cerebral Artery Occlusion

Middle cerebral artery occlusion (MCAO), used for induction of a focal cerebral ischemia, was performed according to the published protocol [16]. In brief, the rats were anesthetized with isoflurane. An incision was made in the neck area and, pushing the muscle tissue on the left side, common carotid artery was reached and ligatures applied to it, as well as to external and internal carotid arteries. A nylon filament (3-0) with a rounded end was inserted through the opening at the bifurcation site onto the external and internal branches and advanced along the internal carotid artery to the middle cerebral artery. Then, the ligature on the internal carotid artery was tightened to fix the filament. Occlusion was performed for 60 min, while common, external, and internal carotid arteries remained ligated, and the body temperature of the animal was maintained at 37 ± 0.5 °C. Then, the filament was removed and the ligature on the internal carotid artery was tightened. In the group of sham operated rats, all manipulations were performed, except for the insertion of the filament. The sham group was used as a control to MCAO. 

The development of ischemic stroke as a result of MCAO was verified by monitoring neurological deficit using two tests 24 h after a surgery. Three MCAO rats and three sham operated rats (body mass 240–274 g) were used for transcriptome analysis. Neurological deficits were assessed using two tests. The five-point behavioral scale [17] allows assessment of the functional status of the contralateral foreleg by watching turns and circulations in the contralateral side, as well as the mobility of animals. The scores were 3, 2 and 3 in MCAO rats (2, decreased grip of right forelimb; 3, circling to contralateral side while tail pulled) vs. 0 in shams. Tongue protrusion test [18] was used for assessment of the stroke-sensitive ability of a rat to lick peanut butter from a glass cylinder, followed by measuring the distance from the beginning of the cylinder to the level of the remaining butter. The measures were 0, 0, and 2 mm vs. 14, 9, and 10 mm in shams. The results of both tests confirmed a significant neurological deficit in MCAO rats as compared to sham operated control. 

### 2.3. LPS Administration

LPS (30 μg in 4 μL of sterile saline) or saline (SAL) were infused stereotactically into the right striatum under isoflurane anesthesia (4% isoflurane for induction, 2.5% for maintenance in O2 at a flow rate of 1 L/min) using the coordinates: AP = + 0.5 mm, ML = + 3 mm, DV = −5.5/4.5 mm [15]. It has been shown in a pilot study using real time PCR that this injection induces a significant increase in expression of interleukin-1β (IL-1β), a pro-inflammatory cytokine, in the ipsilateral hippocampus at 24 h: mRNA expression (fold) for intact animals—1.062 ± 0.146 (8), saline control—0.811 ± 0.144 (8), LPS—9.209 ± 3.441 (7); F(2, 20) = 6.6728, *p* < 0.01; Tukey post hoc indicates *p* < 0.05 for LPS vs. both control groups. 

### 2.4. Collecting Hippocampal Samples

Twenty-four hours after MCAO or LPS administration, the rats were sacrificed by rapid decapitation. Brains were quickly extracted and ipsilateral hippocampi (*n* = 3 for each group of MCAO, SHAM, LPS, SAL) were rapidly isolated and each was placed in an Eppendorf tube with 1 mL of buffer containing an RNase inhibitor (RNAlater) at room temperature. After that, the tube was transferred to ice, after the end of hippocampal collection, stored overnight at +4 °C and then at −80 °C until the analysis of gene expression patterns. 

### 2.5. RNA-Sequencing and Data Analysis

RNA-seq was performed in JSC Genoanalytica (Moscow, Russia; http://genoanalytica.ru accessed on 20 May 2021). For this, total RNA was extracted from ipsilateral hippocampus in ischemic and LPS-infused rats with Trisol reagent according to manufacture instruction. Quality was checked with BioAnalyser and RNA 6000 Nano Kit (Agilent, Santa Clara, CA, USA). PolyA RNA was purified with Dynabeads^®^ mRNA Purification Kit (Ambion, Thermo Fisher Scientific, Waltham, MA, USA). Illumina library was made from polyA NEBNext^®^ Ultra™ II RNA Library Prep (NEB, Ipswich, MA, USA) according to manual. Sequencing was performed on HiSeq1500 with 50 bp read length. At least 10 million reads were generated for each sample. 

The raw reads from RNA-seq experiments were trimmed for quality (phred ≥ 20) and length (bp ≥ 32) using Trimmomatic v3.2.2 [19]. Reads were mapped to the Rnor_6.0 genome with STAR aligner [20] and differentially expressed transcripts were inferred by DESeq2.0 [21]. Genes with an adjusted *p*-value (padj) less than 0.05 were classified as significantly differentially expressed genes (DEGs). 

The Database for Annotation, Visualization and Integrated Discovery (DAVID; version 6.8; https://david.ncifcrf.gov/ accessed on 20 May 2021 [22]) was used to perform functional annotation and functional annotation clustering of DEGs with more than a two-fold change (FC) in expression. For functional annotation, a false discovery rate (FDR) of 0.05 was selected as the cutoff criterion. For functional annotation clustering, following parameters corresponding to a “medium” level of stringency was used: kappa similarity–similarity term overlap: 3, similarity threshold: 0.50; classification—initial group membership: 3, final group membership: 3, multiple linkage threshold: 0.50; enrichment thresholds–EASE: 1.0. 

## 3. Results

Genome-wide transcriptome analysis was performed to reveal the effect of MCAO-induced immune/inflammatory activation on hippocampal global gene expression. For this, we used genes whose expressions were changed after LPS infusion into striatum as the marker genes to extract similar ones from the list of MCAO DEGs. 

### 3.1. DEGs in the Hippocampus of MCAO vs. SHAM in Overall and Similar Response after LPS

Differential expression analysis identified a total of 213 DEGs with an adjusted *p* value (padj) of less than 0.05 and a│log2FC│ > 0.95 in the hippocampus after MCAO as compared with the sham controls (Table S1, Supplementary Materials). Of the 213 transcripts, 182 and 31 were up- and downregulated, respectively. Comparison of these upregulated and downregulated genes with those after LPS infusion into striatum revealed 84 DEGs common to both paradigms (Table 1). Among the commonly changed genes, only one gene (*P2ry12*) was significantly downregulated. 

Tree lists of MCAO DEGs were used for bioinformatics analyses using the DAVID online tool: (a) all DEGs; (b) DEGs that responded to both MCAO and LPS; (c) DEGs that responded to MCAO, but not to LPS.

### 3.2. Gene Ontology (GO) Functional Enrichment Analysis

#### 3.2.1. Biological Process (BP) 

**Figure 1:**
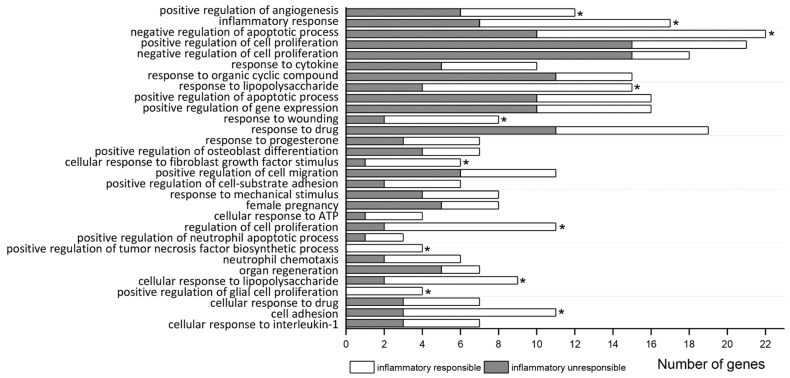
Significant enrichment terms (FDR < 0.05) of GO biological process for all MCAO DEGs. The number of DEGs at each term includes genes responding (white) or not (dark) to inflammatory signal (LPS). Asterisks (*) indicate terms which also significantly enrichment for inflammatory responding genes separately.

As shown in Table 2 and Figure 1, 30 biological process (BP) terms were significantly enriched (FDR < 0.05) in the list of all 213 MCAO DEGs. These GO BPs were related to immune/inflammatory activation (“inflammatory response”, “response to cytokine”, “response to lipopolysaccharide”, “cellular response to fibroblast growth factor stimulus”, “cellular response to ATP”, “positive regulation of tumor necrosis factor biosynthetic process”, “neutrophil chemotaxis”, “cellular response to lipopolysaccharide”, “positive regulation of glial cell proliferation”, “cell adhesion”, “cellular response to interleukin-1”), cell death (“positive regulation of apoptotic process”) and tissue repair (“positive regulation of angiogenesis”, “positive regulation of cell proliferation”). Genes belonging to most terms were from both MCAO groups (responding or not responding to LPS), excluding 2 terms (“positive regulation of tumor necrosis factor biosynthetic process” and “positive regulation of glial cell proliferation”), which were completely enriched only by the LPS-responding genes (Table 2, Figure 1). 

Significance of 11 from 30 BPs related to immune/inflammatory activation as well as to “regulation of cell proliferation”, “negative regulation of apoptotic process”, “response to wounding”, and “positive regulation of angiogenesis” was reached due to DEGs responding to LPS (Table S2a). Analysis of MCAO DEG subgroup that did not respond to LPS revealed their preferential involvement in “negative regulation of cell proliferation”, “positive regulation of cell proliferation”, and also in “response to organic cyclic compound” (Table S2b).

#### 3.2.2. Molecular Function 

In the list of all 213 MCAO DEGs, significantly enriched (FDR < 0.05) molecular function terms were “heparin binding”, “extracellular matrix binding”, and “S100 protein binding” (Table S3a). In the list of DEGs (84) responding to both MCAO and LPS, only 1 significantly enriched (FDR < 0.05) molecular function term, “integrin binding”, was revealed (Table S3b). No significantly enriched (FDR < 0.05) molecular function terms were found in non-responding to LPS genes.

#### 3.2.3. Cellular Component

Ten significantly enriched (FDR < 0.05) cellular component terms were found in the list of all MCAO DEGs (Table S4a). Six significantly enriched (FDR < 0.05) cellular component terms (“extracellular space”, “extracellular exosome”, “extracellular matrix”, “cell surface”, “vesicle”, “external side of plasma membrane”) were the same in the list of genes responding to both MCAO and LPS (Table S4b). Only two significantly enriched (FDR < 0.05) cellular component terms (“extracellular region” and “extracellular space”) were revealed in the list of 129 genes that did not respond to LPS (Table S4c).

### 3.3. Functional Categories (UP_KEYWORDS)

Eight up keywords were significantly enriched (FDR < 0.05) in the list of all MCAO genes (Table S5a). In the lists of commonly responding transcripts and non-responding to LPS ones, 9 (Table S5b) and 4 (Table S5c) up keywords, accordingly, were significantly enriched (FDR < 0.05).

### 3.4. KEGG Pathway Enrichment Analysis

**Figure 2:**
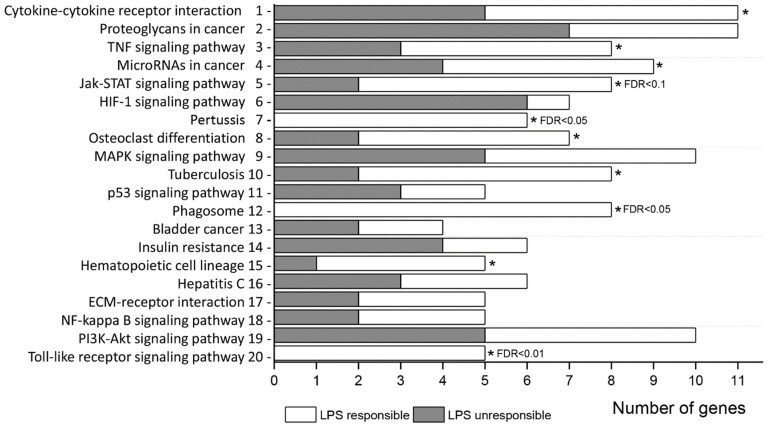
Significant enrichment terms (1–4: FDR < 0.05; 5–7: FDR < 0.1; 8–20: *p* < 0.05) of GO biological process for all MCAO DEGs. The number of DEGs at each term includes genes responding (white) or not (dark) to inflammatory signal (LPS). Asterisks (*) indicate terms which also significantly enrichment (*p* < 0.05 and moreover, FDR < 0.1 or FDR < 0.05) for inflammatory responding genes separately.

To further understand the function of genes responding or not-responding to inflammatory stimuli, KEGG pathway enrichment analysis was performed. All DEGs between MCAO and SHAM control groups were significantly (*p* < 0.05) enriched to 20 pathway terms (Table 3, Figure 2) with maximal enrichment (FDR < 0.05) for “cytokine-cytokine receptor interaction”, “proteoglycans in cancer”, “TNF signaling pathway”, and “MicroRNAs in cancer”. In the list of commonly responding genes, top two pathways with greatest enrichment (FDR < 0.05) were “Pertussis” and “Phagosome” (Table S6a), whereas in the list of non-responding to LPS genes, the top pathway was “HIF-1 signaling pathway” (Table S6b). 

### 3.5. Functional Annotation Clustering

The functional annotation clustering of genes was based on their putative functions revealed from GO, KEGG pathways, and keywords data. For all (213) MCAO DEGs, 26 clusters were identified, of which 11 clusters reached an enrichment score of 1.5 or greater. The cluster 1 was significantly enriched (*p* < 0.05) with terms related to Innate immunity, Immunity and innate immune response (enrichment score: 2.58, 10 genes: *Clec5a, CD14, Fgr, Hck, Anxa1, Lgals3, Irf1, Lbp, Ptx3, Serping1*) (Table S7a).

**Figure 3:**
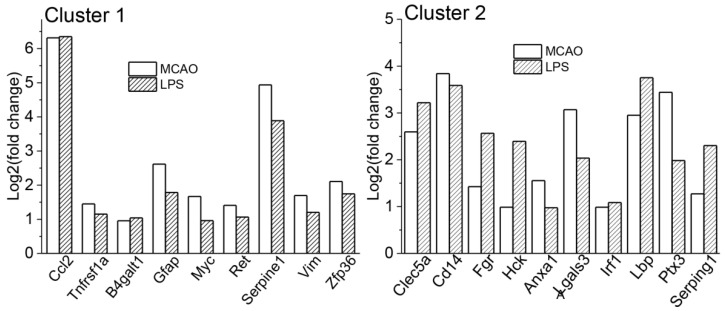
Figure shows DEGs included in clusters 1 and 2 from functional annotation clustering of 84 genes responding to both MCAO and LPS.

The functional annotation clustering of commonly responding genes (84) generated 18 clusters, of which 10 clusters reached an enrichment score of 1.5 or higher. Two top clusters (Table 4) with the highest significance included terms related to response to wounding, cellular response to fibroblast growth factor stimulus, MAPK cascade (Cluster 1, enrichment score: 3.63, 9 genes), innate immunity, immunity, and innate immune response (Cluster 2, enrichment score: 3.39, 10 genes). No overlap was observed between the genes in each of these two clusters (Table 4, Figure 3). Importantly, the set of genes from cluster 2 presented on Figure 3 was completely the same as in cluster 1 for all (213) MCAO genes as well as for all upregulated (182) MCAO genes (Table S7b). 

Functional annotation clustering of 129 non-responding to LPS MCAO-related genes revealed 14 clusters, of which only two clusters reached an enrichment score of 1.5 or higher. These clusters included terms related to secreted/signal/extracellular region/extracellular space/disulfide bond (cluster1, enrichment score: 3.72, 42 genes), growth factor/growth factor activity/cytokine activity (cluster2, enrichment score: 1.72, 6 genes).

## 4. Discussion

### 4.1. All Genes That Were Involved in Immune Activation after MCAO Responded to LPS

Neuroinflammation is believed to contribute to the delayed stroke-induced cell death in the hippocampus observed already 24 h after cerebral ischemia [23,24]. Acute alterations in gene expression can be important in stimulation of this consequence of ischemia [25]. Using MCAO to model the focal ischemia in rats and RNA-seq to identify DEGs, we aimed to further clarify molecular mechanisms mediating the pathways of the immunity/inflammatory activation in the hippocampal structure. The main finding of the present study is that 10 genes, *Clec5a*, *CD14*, *Fgr*, *Hck*, *Anxa1*, *Lgals3*, *Irf1*, *Lbp*, *Ptx3*, and *Serping1*, may represent key molecular factors of the acute activation of immune cells in the hippocampus in response to MCAO. Functional annotation clustering showed that same set of genes responding to direct pro-inflammatory stimulus included in the clusters related to innate immunity/immunity/innate immune response in both all (213 genes) MCAO DEGs (cluster 1) and in responding to LPS MCAO subgroup (84 DEGs; cluster 2). 

The first immune cells responding to ischemic insults are the microglial cells [26]. Their activation triggers the inflammatory responses which are mediated by cytokines, chemokines, and growth factors produced by numerous cells including, in addition to resident macrophages, infiltrated leukocytes and also the non-immune cells such as endothelial cells and fibroblasts. Activation of the microglial cells can be induced by various pathogen- and damage-associated molecular patterns—PAMPs and DAMPs, respectively [27].

Toll-like receptors (Tlrs) play an important role in PAMPs recognition. Among the 10 genes mentioned above, the increase in expression of *Lbp* (lipopolysaccharide binding protein), *Cd14* (CD14 molecule), and *Lgals3* (galectin 3) is likely related to an activation of microglial cells through Tlrs. An initiation of the Tlr signaling can occur through transfer of bacterial LPS, particularly as plasma levels of LPS are elevated in ischemic stroke [5,28], by Lbp to a receptor complex that localizes on microglia and includes Tlrs (the most known Tlr4) and co-receptor CD14. Expression of another gene, *Lgals3*, was related to microglial activation in experimental ischemia in gerbils [29]. Protein product of this gene released by microglia can act as an endogenous paracrine Tlr4 ligand prolonging the inflammatory response in the brain [30]. In addition to innate immunity, Lgals3 participates in cell adhesion and neuro-vascular protection through modulation of angiogenic and apoptotic pathways [31]. In our study, *Lgals3* was associated with BPs such as “neutrophil chemotaxis”, “positive regulation of angiogenesis”, “negative regulation of apoptotic process”, and “positive regulation of cell proliferation”. 

Pentraxin 3 (*Ptx3*) regulates neutrophil transmigration in the brain [32]. Mobilization of innate immunity includes development of complement cascade evidenced in our study by elevated expression of *Serping1* (serpin family G member 1). Its protein product, C1 inhibitor, is believed to play anti-inflammatory and anti-thrombotic roles and protect from ischemic neurodegeneration [33]. *Clec5a* (also known as myeloid DAP12-associating lectin; *MDL-1*) has been implicated in activation of myeloid cells, particularly monocytes, macrophages, and neutrophils [34] and recently suggested as a potential therapeutic target for attenuation of both septic and aseptic inflammatory reactions [35]. Myeloid-specific Src family kinases, *Fgr* and *Hck*, participate in the initiation of phagocytosis and secretion of cytokines by activated macrophages [36]. *Anxa1* (annexin A1) plays a role in the microglial clearance of apoptotic neurons in both non-inflammatory and inflammatory conditions [37]. *Irf1* (interferon regulatory factor 1) is an important transcription factor implicated in downstream Tlr signaling [38]. 

### 4.2. Inflammatory Response

The recognition of pathogens, including endotoxin, can lead to inflammatory activation starting with the increase in expression of proinflammatory cytokine genes, in particular *TNFα* [39]. All MCAO up-regulated DEGs with functional implication in “toll-like receptor signaling pathway” (KEGG pathway): *Cd14*, *Fos*, *Lbp*, *Spp1*, *Tlr1*, or BP “positive regulation of tumor necrosis factor biosynthetic process”: *Hspb1*, *Lbp*, *Thbs1*, *Tlr1*, also responded to LPS. 

TNFα is the proinflammatory cytokine exerting major effects on the hippocampal production of other inflammatory factors, e.g., IL-1β [40] and chemokines [41]. In our study, expression of TNF receptors upregulated in the hippocampus after both LPS and MCAO included *Tnfrsf1a*, *Tnfrsf12a*, and *Tnfrsf26*. The increase in *Tnfrsf1a* expression in the hippocampus of rats 24 h after ischemia was reported previously [11], and our data support this result. GO BP terms “inflammatory response”, “response to lipopolysaccharide”, and “regulation of cell proliferation” were mainly enriched with LPS-responding genes, including *Tnfrsf1a* and *Tnfrsf26*, and the numbers of these LPS-responding genes were 10 (*Ccl2*, *Cd14*, *Hck*, *Tnfrsf1a*, *Anxa1*, *Casp4*, *Spp1*, *S1pr3*, *Thbs1*, *Tnfrsf26*) from 17, 11 (*Ccl2*, *Cd14*, *Fos*, *Tnfrsf1a*, *Csf2rb*, *Lbp*, *Ptges*, *Serpine1*, *Socs3*, *Trib1*, *Tnfrsf26*) from 15, and 9 (*Fgr*, *Hck*, *S100a11*, *Tnfrsf1a*, *Anxa1*, *B4galt1*, *Irf1*, *Serpine1*, *Tnfrsf26*) from 11, accordingly. Furthermore, GO analysis showed significance of these GO BP terms enriched only with LPS-responding genes. In addition, “TNF signaling pathway” was one of the top enriched pathways and five (*Bcl3*, *Ccl2*, *Fos*, *Tnfrsf1a*, *Socs3*) of eight DEGs associated with this pathway were LPS-dependent, which also significantly enriched this pathway in separate analysis. 

The key participants of inflammatory activation are chemokines and adhesion molecules playing an important role in leukocyte infiltration. GO enrichment analysis revealed a variety of such genes, LPS-responding and not-responding, for example, *Ccl2*, *Itga5*, *Itgam and Ccl7*, *Cxcl12*, *Itgax*, accordingly, belonging to BP terms “neutrophil chemotaxis” (*Ccl2*; *Ccl7*, *Itgam*), “cell adhesion” (*Itgax*, *Itga5*, *Itgam*), “positive regulation of cell-substrate adhesion” (*Itga5*). 

The increase in nucleotide release resulting from brain injury was shown to be involved in activation of microglial cells as well as in their chemotaxis by acting on purinergic receptor P2Y12 (P2ry12) [42]; the expression of *P2ry12* was downregulated in response to both MCAO and LPS in our study. Significant decrease in hippocampal *P2ry12* expression was found previously after microglial activation by systemic LPS administration [43]. It has been reported that P2Y12 receptor activation on microglia aggravates ischemic stroke injury [44]. Four DEGs, including LPS-responding *Ccl2, P2ry12*, and *Serpine1* (serpin family E member 1), and non-responding to LPS *Ptgs2*, were enriched in GO BP term “cellular response to ATP”.

### 4.3. DEGs Associated with Apoptotic Processes

The most upregulated chemokine gene in response to both MCAO and LPS was *Ccl2*. A significant elevation in *Ccl2* mRNA expression in the rat hippocampus was found already 6 h after the ischemia/reperfusion [45]. Increased expression of *Ccl2* was associated with neuronal death: intrahippocampal infusions of Ccl2 induced apoptosis in hippocampal neurons [46]. Mechanisms of cerebral stroke-induced hippocampal injury and neuroinflammation development may include glutamate excitotoxicity caused by a significant rise in extracellular level of glutamate in the hippocampus [47]. NMDA-induced neuronal injury resulted in astrocytic CCL2 production in rat corticostriatal slice cultures [48].

Excitotoxic death in hippocampal neurons can be further augmented by glucocorticoids secreted as a result of ischemia-induced hypothalamic–pituitary–adrenal axis activation [49]. Glucocorticoid stress hormones predominantly stimulated anti-inflammatory responses however some reports prove a glucocorticoid-dependent augmentation of inflammation after ischemia. For example, a pro-inflammatory effect was revealed in the hippocampus of rats 24 h after MCAO; this effect was mediated by glucocorticoid receptors of myeloid and endothelial cells [50]. Onufriev et al. [51] demonstrated accumulation of corticosterone and IL-1β in the hippocampus of rats during the acute period after MCAO, suggesting the association of glucocorticoid excess with neuroinflammation. These data indicate a dual role of glucocorticoids in hippocampal neuroinflammation (either anti- or pro-inflammatory), though conditions promoting these opposite situations—as well as gene-related mechanisms—remain obscure [52]. Using dexametasone as a reference glucocorticoid, Tret’yakova et al. demonstrated that, injected intrahippocampally, it was able to induce only weak neuroinflammation, but when applied during LPS-induced neuroinflammation, dexametasone significantly influenced cytokine balance in the hippocampus [53]. Stress-related gene *Hspb1* upregulated after both MCAO and LPS in our study was found among the key genes correlated with ischemic injury [54]. 

Two genes, *Ptgs2* (prostaglandin-endoperoxide synthase 2; cyclooxygenase 2) and *Ptges* (prostaglandin E synthase), encoding the key enzymes in prostaglandin E2 (PgE2) synthesis, were significantly upregulated by MCAO, and, in addition, *Ptges* expression was also increased by LPS. Previously, significant increase in Ptgs2 protein expression was observed in the rat hippocampus 12 h, 24 h, and 48 h after MCAO [55]. PgE2 was also suggested as an inflammation mediator which accelerates microglial activation and peripheral immune cell infiltration resulting in augmentation of ischemic brain damage. Therefore, differences in sensitivity of synthesis enzyme expression to environmental factors may be involved in pro-inflammatory and damaging effects of PgE2. For example, genetic deletion of *Ptgs2* or post-ischemic treatment with its inhibitor significantly reduced ischemic stroke injury [56]. Damaging effects of PgE2 are associated with increase in matrix metalloproteinase expression via TNF-signaling pathway [57]. Increased expression of *Adamts1* (ADAM metallopeptidase with thrombospondin type 1 motif 1) encoded protease that is involved in extracellular matrix proteolysis, and two TNF receptors, *Tnfrsf1a* and *Tnfrsf26*, also supports an association of these genes with inflammatory activation and hippocampal tissue injury.

GO enrichment analysis for all MCAO DEGs revealed that 16 genes are involved in BP “positive regulation of apoptotic process”. Among these genes, 6 genes (*Tnfrsf12a*, *Anxa1*, *B4galt1*, *Cyr61*, *Tgm2*, *Tspo*) responded to LPS, and 10 genes, 8 upregulated (*Fosl1*, *Adm*, *Gadd45a*, *Gadd45g*, *Hmox1*, *Pawr*, *Ptgs2*, *Ppp1r15a*), and 2 downregulated (*Frzb*, *Sfrp2*) did not respond. As for LPS-responding genes, *Tnfrsf12a* also known as fibroblast growth factor-inducible immediate-early response protein 14, is a cell surface receptor for tumor necrosis factor-like weak inducer of apoptosis (TWEAK). Both these factors are expressed in neurons and involved in neuronal death mediated by nuclear factor-kappa B (NF-κB) pathway activation [58]. They were upregulated in the infarcted and peri-infarct brain tissue in stroke patients within 24 h [59]. *Anxa1* (Annexin A1) was shown to have multiple functions, and taking into account its anti-inflammatory effects, it was suggested to be neuroprotective during ischemic stroke injury [60]. *Tgm2* (Transglutaminase 2) is involved in many biological processes, including control of apoptotic cell clearance by macrophages [61,62]. Non-responding to LPS, *Gadd45a* (Growth arrest and DNA damage inducible alpha) plays a role in mediating glutamate-induced oxidative cytotoxicity in hippocampal neuronal cells [63]. 

### 4.4. DEGs Associated with Recovering and Neuroprotective Processes

Clearance of damaged cells by macrophages is important for recovery of the normal tissue homeostasis [64]. Herein we found that all MCAO genes related to KEGG pathway “Phagosome” were LPS-responding: *Cd14*, *Fcgr2b*, *Itga5*, *Itgam*, *Msr1*, *Ncf4*, *Thbs1*, *Tubb6*. 

Biological process “positive regulation of angiogenesis” was equally enriched with LPS-responding and non-responding genes; however, “positive regulation of cell proliferation” was enriched mainly with non-responding ones (15 from 21). Three of six LPS-responding genes—*Il11*, *Myc*, and *Osmr*—also related to “Jak-STAT signaling pathway”, whereas only two genes—*Lif* and *Stat3*—from non-responding subgroup were related to this KEGG pathway. In cardiomyocytes, Il-11 promoted cell survival and angiogenesis through the JAK/STAT pathway in ischemia/reperfusion model [65]. Treatment with IL-11 inhibited apoptosis and reduced cerebral infarct volume induced by MCAO [66]. In our study, transcriptional factor *Stat3* was also associated with “positive regulation of gene expression”, “response to drug”, “response to cytokine”, “negative regulation of cell proliferation”, and “negative regulation of apoptotic process”.

Astrocytes are believed to be involved in acute immune defense against pathogens [67]. This suggestion is based on their ability to maintain the blood-brain-barrier integrity, release brain-derived neurotrophic factor and take up excessive glutamate from extracellular space [41]. An increase in expression of *Gfap* (glial fibrillary acidic protein), a marker of astrocytes, indicates an activation of these glial cells after both MCAO and LPS exposures. Twenty-four hours after ischemia, *Gfap* was among genes that enriched BP “response to wounding”. 

Together, our findings suggest that hippocampal genes responding to bacterial infection are associated with acute neuroinflammatory responses to cerebral ischemia that may be implicated in provocation of delayed changes in the brain structure. Understanding the mechanisms of such a possible provocative action requires further investigations using long-term survival ischemia models. For example, global cerebral ischemia performed by cardiac arrest of 10 min duration caused neuroinflammatory reaction in the rat hippocampus even 2 years after the insult [68]. This type of brain ischemia also activated neuronal changes and death in the CA3 region of the hippocampus in a manner dependent on amyloid and tau protein [69].

## 5. Conclusions

**Figure 4:**
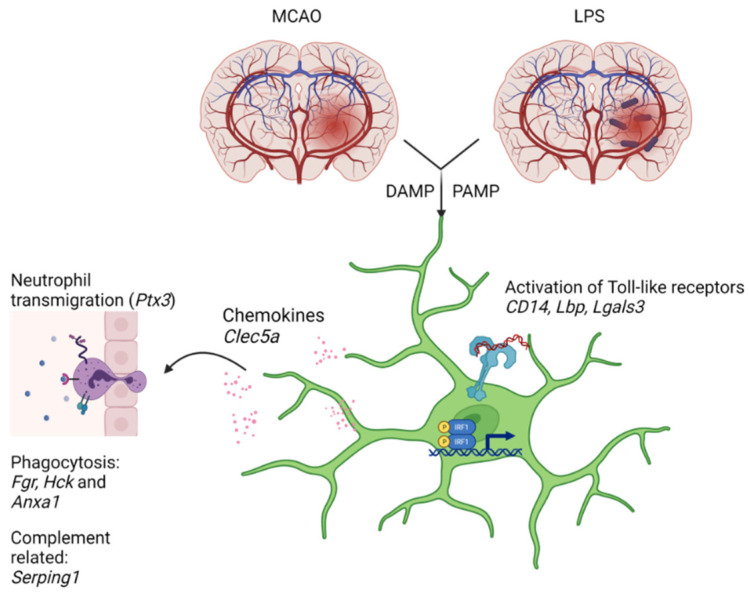
A scheme illustrating the processes related to 10 genes: *Clec5a*, *CD14*, *Fgr*, *Hck*, *Anxa1*, *Lgals3*, *Irf1*, *Lbp*, *Ptx3*, *Serping1*. Variety of types of signals including DAMPs (damage-associated molecular patterns such as proteins and nucleotides from damaged or dying cells), PAMPs (pathogen-associated molecular patterns such as endotoxins), glutamate and glucocorticoids activate microglia by multiple mechanisms, among which are Toll-like receptors (Tlr) (*CD14, Lbp)*, and their downstream signaling (*Irf1*). Galectin 3 (*Lgals3*) can work as an endogenous Tlr ligand. Activated microglia released different cytokines and chemokines, which implicated in regulation of neutrophil activation and transmigration in the brain (*Ptx3*, *Clec5a*). Increased expression of *Fgr*, *Hck*, and *Anxa1* was associated with initiation of phagocytosis, whereas an increase in *Serping1* expression may reflect complement cascade development.

The results received in the present study suggest that alterations in expression of 10 genes (*Clec5a*, *CD14*, *Fgr*, *Hck*, *Anxa1*, *Lgals3*, *Irf1*, *Lbp*, *Ptx3*, *Serping1*) responding to direct pro-inflammatory stimuli such as LPS may contribute to molecular mechanisms of the acute MCAO-induced activation of immune cells in the hippocampus. Figure 4 shows a scheme illustrating the processes related to these 10 genes. 

## Figures and Tables

**Table 1:** List of 84 DEGs that were common among MCAO vs. SHAM and LPS vs. SAL at 24 h.

*n*	Ensembl Gene ID	Gene Symbol	Gene Description	MCAO vs. SHAM			LPS vs. SAL		
				log2 FC	*p*-Value	padj	log2 FC	*p*-Value	padj
1	ENSRNOG00000007159	*Ccl2*	C-C motif chemokine ligand 2	6.32	1.50 × 10^−13^	1.20 × 10^−10^	6.35	5.00 × 10^−5^	2.21 × 10^−3^
2	ENSRNOG00000043451	*Spp1*	Secreted phosphoprotein 1	6.11	5.10 × 10^−10^	2.20 × 10^−7^	3.73	5.00 × 10^−5^	2.21 × 10^−3^
3	ENSRNOG00000002918	*FAM187A*	Family with sequence similarity 187, member A	5.62	5.40 × 10^−10^	2.30 × 10^−7^	2.60	5.00 × 10^−5^	2.21 × 10^−3^
4	ENSRNOG00000001414	*Serpine1*	Serpin family E member 1	4.94	8.50 × 10^−8^	2.00 × 10^−5^	3.89	5.00 × 10^−5^	2.21 × 10^−3^
5	ENSRNOG00000023546	*Hspb1*	Heat shock protein family B (small) member 1	4.55	6.90 × 10^−6^	8.50 × 10^−4^	2.72	5.00 × 10^−5^	2.21 × 10^−3^
6	ENSRNOG00000002946	*Socs3*	Suppressor of cytokine signaling 3	4.30	4.40 × 10^−24^	1.60 × 10^−20^	2.82	5.00 × 10^−5^	2.21 × 10^−3^
7	ENSRNOG00000017819	*Cd14*	CD14 molecule	3.84	5.50 × 10^−18^	1.10 × 10^−14^	3.59	5.00 × 10^−5^	2.21 × 10^−3^
8	ENSRNOG00000000187	*Csf2rb*	Colony stimulating factor 2 receptor beta common subunit	3.69	8.20 × 10^−33^	1.20 × 10^−28^	4.68	5.00 × 10^−5^	2.21 × 10^−3^
9	ENSRNOG00000012843	*Aspg*	Asparaginase	3.48	4.50 × 10^−11^	2.50 × 10^−8^	2.30	5.00 × 10^−5^	2.21 × 10^−3^
10	ENSRNOG00000012280	*Ptx3*	Pentraxin 3	3.44	9.80 × 10^−6^	1.10 × 10^−3^	1.98	5.00 × 10^−5^	2.21 × 10^−3^
11	ENSRNOG00000003745	*Atf3*	Activating transcription factor 3	3.25	1.70 × 10^−12^	1.20 × 10^−9^	2.06	2.50 × 10^−4^	9.30 × 10^−3^
12	ENSRNOG00000017386	*Il11*	Interleukin 11	3.07	1.20 × 10^−5^	1.40 × 10^−3^	1.84	5.00 × 10^−5^	2.21 × 10^−3^
13	ENSRNOG00000010645	*Lgals3*	Galectin 3	3.07	5.60 × 10^−8^	1.40 × 10^−5^	2.04	5.00 × 10^−5^	2.21 × 10^−3^
14	ENSRNOG00000043416	*Bcl3*	B-cell CLL/lymphoma 3	3.06	1.10 × 10^−13^	9.70 × 10^−11^	3.43	5.00 × 10^−5^	2.21 × 10^−3^
15	ENSRNOG00000014838	*Glipr2*	GLI Pathogenesis Related 2	3.04	2.60 × 10^−9^	8.90 × 10^−7^	2.32	5.00 × 10^−5^	2.21 × 10^−3^
16	ENSRNOG00000014532	*Lbp*	Lipopolysaccharide binding protein	2.95	2.80 × 10^−4^	1.50 × 10^−2^	3.75	5.00 × 10^−5^	2.21 × 10^−3^
17	ENSRNOG00000012886	*Maff*	MAF bZIP transcription factor F	2.91	1.50 × 10^−22^	4.30 × 10^−19^	2.54	5.00 × 10^−5^	2.21 × 10^−3^
18	ENSRNOG00000012779	*Msr1*	Macrophage scavenger receptor 1	2.84	2.00 × 10^−4^	1.20 × 10^−2^	4.52	5.00 × 10^−5^	2.21 × 10^−3^
19	ENSRNOG00000003546	*Tnfrsf12a*	TNF receptor superfamily member 12A	2.77	1.30 × 10^−9^	4.90 × 10^−7^	1.14	6.00 × 10^−4^	1.95 × 10^−2^
20	ENSRNOG00000002919	*Gfap*	Glial fibrillary acidic protein	2.62	3.50 × 10^−7^	6.70 × 10^−5^	1.79	5.00 × 10^−5^	2.21 × 10^−3^
21	ENSRNOG00000026306	*Clec5a*	C-type lectin domain family 5, member A	2.60	6.10 × 10^−5^	4.40 × 10^−3^	3.22	5.00 × 10^−5^	2.21 × 10^−3^
22	ENSRNOG00000006320	*Ptges*	Prostaglandin E synthase	2.44	2.90 × 10^−7^	5.70 × 10^−5^	2.90	5.00 × 10^−5^	2.21 × 10^−3^
23	ENSRNOG00000008015	*Fos*	FBJ osteosarcoma oncogene	2.42	3.50 × 10^−27^	2.50 × 10^−23^	2.47	5.00 × 10^−5^	2.21 × 10^−3^
24	ENSRNOG00000010513	*Tfpi2*	Tissue factor pathway inhibitor 2	2.39	2.50 × 10^−7^	5.30 × 10^−5^	1.56	7.00 × 10^−4^	2.21 × 10^−2^
25	ENSRNOG00000012881	*Fgl2*	Fibrinogen-like 2	2.38	1.30 × 10^−16^	1.90 × 10^−13^	1.70	5.00 × 10^−5^	2.21 × 10^−3^
26	ENSRNOG00000010105	*S100a11*	S100 calcium binding protein A11	2.38	1.50 × 10^−5^	1.50 × 10^−3^	1.26	5.00 × 10^−5^	2.21 × 10^−3^
27	ENSRNOG00000033192	*Osmr*	Oncostatin M receptor	2.37	5.40 × 10^−17^	8.50 × 10^−14^	1.08	1.50 × 10^−4^	5.95 × 10^−3^
28	ENSRNOG00000058568	*Dhrs9*	Dehydrogenase/reductase 9	2.31	3.20 × 10^−6^	4.20 × 10^−4^	1.54	5.00 × 10^−5^	2.21 × 10^−3^
29	ENSRNOG00000018371	*Tubb6*	Tubulin, beta 6 class V	2.21	4.90 × 10^−4^	2.30 × 10^−2^	1.19	5.00 × 10^−5^	2.21 × 10^−3^
30	ENSRNOG00000014524	*S1pr3*	Sphingosine-1-phosphate receptor 3	2.21	9.10 × 10^−11^	4.30 × 10^−8^	1.48	5.00 × 10^−5^	2.21 × 10^−3^
31	ENSRNOG00000001607	*Adamts1*	ADAM metallopeptidase with thrombospondin type 1 motif, 1	2.15	8.70 × 10^−21^	2.10 × 10^−17^	1.30	5.00 × 10^−5^	2.21 × 10^−3^
32	ENSRNOG00000010549	*Tspo*	Translocator protein	2.15	1.90 × 10^−5^	1.90 × 10^−3^	2.96	5.00 × 10^−5^	2.21 × 10^−3^
33	ENSRNOG00000006940	*Ncf4*	Neutrophil cytosolic factor 4	2.13	2.70 × 10^−6^	3.70 × 10^−4^	3.01	5.00 × 10^−5^	2.21 × 10^−3^
34	ENSRNOG00000008215	*Trim47*	Tripartite motif containing 47	2.12	7.60 × 10^−14^	7.20 × 10^−11^	1.21	5.00 × 10^−5^	2.21 × 10^−3^
35	ENSRNOG00000058388	*Zfp36*	Zinc finger protein 36	2.11	3.70 × 10^−26^	1.80 × 10^−22^	1.75	5.00 × 10^−5^	2.21 × 10^−3^
36	ENSRNOG00000046452	*Fcgr2b*	Fc fragment of IgG, low affinity IIb, receptor	1.94	6.10 × 10^−9^	2.00 × 10^−6^	1.53	5.00 × 10^−5^	2.21 × 10^−3^
37	ENSRNOG00000004273	*Ifitm1*	Interferon induced transmembrane protein 1	1.86	3.10 × 10^−5^	2.70 × 10^−3^	2.70	5.00 × 10^−5^	2.21 × 10^−3^
38	ENSRNOG00000000878	*Slc44a4*	Solute carrier family 44, member 4	1.85	8.00 × 10^−4^	3.20 × 10^−2^	1.78	5.00 × 10^−5^	2.21 × 10^−3^
39	ENSRNOG00000000529	*Pim1*	Pim-1 proto-oncogene, serine/threonine kinase	1.82	3.90 × 10^−8^	1.00 × 10^−5^	0.95	4.50 × 10^−4^	1.52 × 10^−2^
40	ENSRNOG00000043098	*Mt2A*	Metallothionein 2A	1.81	9.20 × 10^−9^	2.90 × 10^−6^	1.53	5.00 × 10^−5^	2.21 × 10^−3^
41	ENSRNOG00000038722	*Tlr1*	Toll-like receptor 1	1.77	4.70 × 10^−4^	2.30 × 10^−2^	2.54	5.00 × 10^−5^	2.21 × 10^−3^
42	ENSRNOG00000050869	*Cebpd*	CCAAT/enhancer binding protein delta	1.76	2.50 × 10^−17^	4.50 × 10^−14^	1.41	5.00 × 10^−5^	2.21 × 10^−3^
43	ENSRNOG00000004100	*Trib1*	Tribbles pseudokinase 1	1.76	1.80 × 10^−9^	6.40 × 10^−7^	1.03	5.00 × 10^−5^	2.21 × 10^−3^
44	ENSRNOG00000043486	*Tnfrsf26*	Tumor necrosis factor receptor superfamily, member 26	1.73	5.50 × 10^−5^	4.10 × 10^−3^	1.65	1.00 × 10^−4^	4.15 × 10^−3^
45	ENSRNOG00000008816	*Gpnmb*	Glycoprotein nmb	1.70	1.90 × 10^−8^	5.50 × 10^−6^	2.15	5.00 × 10^−5^	2.21 × 10^−3^
46	ENSRNOG00000018087	*Vim*	Vimentin	1.70	1.10 × 10^−3^	3.90 × 10^−2^	1.20	5.00 × 10^−5^	2.21 × 10^−3^
47	ENSRNOG00000004500	*Myc*	Myelocytomatosis oncogene	1.67	4.10 × 10^−12^	2.80 × 10^−9^	0.96	8.50 × 10^−4^	2.58 × 10^−2^
48	ENSRNOG00000005935	*A3galt2*	Alpha 1,3-galactosyltransferase 2	1.66	4.70 × 10^−7^	8.60 × 10^−5^	1.35	5.00 × 10^−5^	2.21 × 10^−3^
49	ENSRNOG00000012865	*Parp3*	Poly (ADP-ribose) polymerase family, member 3	1.58	3.00 × 10^−5^	2.60 × 10^−3^	1.06	5.00 × 10^−5^	2.21 × 10^−3^
50	ENSRNOG00000017469	*Anxa1*	Annexin A1	1.55	2.80 × 10^−5^	2.50 × 10^−3^	0.98	4.00 × 10^−4^	1.37 × 10^−2^
51	ENSRNOG00000019141	*Ch25h*	Cholesterol 25-hydroxylase	1.54	7.10 × 10^−7^	1.20 × 10^−4^	2.01	5.00 × 10^−5^	2.21 × 10^−3^
52	ENSRNOG00000013668	*Capg*	Capping actin protein, gelsolin like	1.53	1.40 × 10^−3^	4.90 × 10^−2^	1.94	5.00 × 10^−5^	2.21 × 10^−3^
53	ENSRNOG00000036677	*Slc16a3*	Solute Carrier Family 16 Member 3	1.50	2.60 × 10^−5^	2.30 × 10^−3^	1.68	5.00 × 10^−5^	2.21 × 10^−3^
54	ENSRNOG00000036834	*Gpr84*	G protein-coupled receptor 84	1.49	2.20 × 10^−7^	4.70 × 10^−5^	1.77	5.00 × 10^−5^	2.21 × 10^−3^
55	ENSRNOG00000045829	*Thbs1*	Thrombospondin 1	1.49	1.50 × 10^−6^	2.40 × 10^−4^	1.41	5.00 × 10^−5^	2.21 × 10^−3^
56	ENSRNOG00000057153	*Pla1a*	Phospholipase A1 member A	1.46	2.50 × 10^−7^	5.30 × 10^−5^	1.70	5.00 × 10^−5^	2.21 × 10^−3^
57	ENSRNOG00000031312	*Tnfrsf1a*	TNF receptor superfamily member 1A	1.45	6.80 × 10^−14^	6.90 × 10^−11^	1.15	5.00 × 10^−5^	2.21 × 10^−3^
58	ENSRNOG00000016413	*Pstpip1*	Proline-serine-threonine phosphatase interacting protein 1	1.43	1.10 × 10^−4^	7.40 × 10^−3^	1.84	5.00 × 10^−5^	2.21 × 10^−3^
59	ENSRNOG00000009912	*Fgr*	FGR proto-oncogene, Src family tyrosine kinase	1.43	3.40 × 10^−5^	2.90 × 10^−3^	2.56	5.00 × 10^−5^	2.21 × 10^−3^
60	ENSRNOG00000014751	*Ret*	Ret proto-oncogene	1.41	2.20 × 10^−8^	6.10 × 10^−6^	1.07	5.00 × 10^−5^	2.21 × 10^−3^
61	ENSRNOG00000033697	*Casp4*	Caspase 4	1.37	8.70 × 10^−6^	1.00 × 10^−3^	1.27	5.00× 10^−5^	2.21 × 10^−3^
62	ENSRNOG00000057451	*Itga5*	Integrin subunit alpha 5	1.36	4.50 × 10^−5^	3.60 × 10^−3^	1.17	5.00 × 10^−5^	2.21 × 10^−3^
63	ENSRNOG00000019728	*Itgam*	Integrin subunit alpha M	1.34	3.80 × 10^−5^	3.10 × 10^−3^	2.30	5.00 × 10^−5^	2.21 × 10^−3^
64	ENSRNOG00000029682	*Clic1*	Chloride intracellular channel 1	1.30	1.60 × 10^−6^	2.40 × 10^−4^	1.62	5.00 × 10^−5^	2.21 × 10^−3^
65	ENSRNOG00000007545	*Angptl4*	Angiopoietin-like 4	1.29	6.90 × 10^−7^	1.20 × 10^−4^	0.99	5.00 × 10^−5^	2.21 × 10^−3^
66	ENSRNOG00000026653	*Hcar2*	Hydroxycarboxylic acid receptor 2	1.28	1.30 × 10^−3^	4.50 × 10^−2^	2.36	5.00 × 10^−5^	2.21 × 10^−3^
67	ENSRNOG00000007457	*Serping1*	Serpin family G member 1	1.27	7.60 × 10^−5^	5.30 × 10^−3^	2.30	5.00 × 10^−5^	2.21 × 10^−3^
68	ENSRNOG00000000827	*Ier3*	Immediate early response 3	1.26	3.20 × 10^−9^	1.10 × 10^−6^	1.23	5.00 × 10^−5^	2.21 × 10^−3^
69	ENSRNOG00000019810	*Des*	Desmin	1.24	3.10 × 10^−4^	1.70 × 10^−2^	1.56	5.00 × 10^−5^	2.21 × 10^−3^
70	ENSRNOG00000011913	*Cp*	Ceruloplasmin	1.24	3.80 × 10^−7^	7.10 × 10^−5^	2.06	5.00 × 10^−5^	2.21 × 10^−3^
71	ENSRNOG00000014258	*Rab32*	RAB32, member RAS oncogene family	1.23	2.20 × 10^−4^	1.30 × 10^−2^	2.04	5.00 × 10^−5^	2.21 × 10^−3^
72	ENSRNOG00000005214	*Plek*	Pleckstrin	1.23	2.20 × 10^−4^	1.30 × 10^−2^	2.29	1.10 × 10^−3^	3.17× 10^−2^
73	ENSRNOG00000005695	*Mgp*	Matrix Gla protein	1.22	8.30 × 10^−13^	6.30 × 10^−10^	1.33	5.00 × 10^−5^	2.21 × 10^−3^
74	ENSRNOG00000008301	*Tagln2*	Transgelin 2	1.20	2.80 × 10^−7^	5.70 × 10^−5^	1.04	5.00 × 10^−5^	2.21 × 10^−3^
75	ENSRNOG00000012956	*Tgm2*	Transglutaminase 2	1.19	1.30 × 10^−5^	1.40 × 10^−3^	1.65	5.00 × 10^−5^	2.21 × 10^−3^
76	ENSRNOG00000004972	*Upp1*	Uridine phosphorylase 1	1.19	1.70 × 10^−5^	1.70 × 10^−3^	1.41	5.00 × 10^−5^	2.21 × 10^−3^
77	ENSRNOG00000014350	*Cyr61*	Cellular communication network factor 1	1.18	8.60 × 10^−4^	3.30 × 10^−2^	1.53	5.00 × 10^−5^	2.21 × 10^−3^
78	ENSRNOG00000015948	*Slc1a5*	Solute carrier gamily 1 member 5	1.13	5.30 × 10^−4^	2.40 × 10^−2^	1.40	5.00 × 10^−5^	2.21 × 10^−3^
79	ENSRNOG00000030118	*Msn*	Moesin	1.05	1.70 × 10^−4^	1.00 × 10^−2^	1.01	5.00 × 10^−5^	2.21 × 10^−3^
80	ENSRNOG00000010362	*Anxa2*	Annexin A2	1.05	1.20 × 10^−3^	4.50 × 10^−2^	0.98	5.00 × 10^−5^	2.21 × 10^−3^
81	ENSRNOG00000008144	*Irf1*	Interferon regulatory factor 1	0.99	8.00 × 10^−5^	5.50 × 10^−3^	1.09	5.00 × 10^−5^	2.21 × 10^−3^
82	ENSRNOG00000009331	*Hck*	HCK proto-oncogene, Src family tyrosine kinase	0.99	1.80 × 10^−4^	1.10 × 10^−2^	2.39	5.00 × 10^−5^	2.21 × 10^−3^
83	ENSRNOG00000059461	*B4galt1*	Beta-1,4-galactosyltransferase 1	0.96	2.10 × 10^−4^	1.20 × 10^−2^	1.04	5.00 × 10^−5^	2.21 × 10^−3^
84	ENSRNOG00000013902	*P2ry12*	Purinergic receptor P2Y12	−1.07	5.10 × 10^−6^	6.40 × 10^−4^	−1.09	9.00 × 10^−4^	2.71 × 10^−2^

**Table 2:** Enriched GO biological process terms for all (213) MCAO DEGs.

	Term	*n*	*p*-Value	FDR	MCAO Genes That Did not Respond to LPS	MCAO Genes That Responded to LPS
1	GO:0045766 positive regulation of angiogenesis	12	1.00 × 10^−7^	1.60 × 10^−4^	6: *Adm, Angpt2, Fgf1, Hmox1, Sfrp2, Sphk1*	6: *Tnfrsf1a, Cyr61, Lgals3, Hspb1, Serpine1, Thbs1*
2	GO:0006954 inflammatory response	17	3.40 × 10^−7^	2.70 × 10^−4^	7: *Ccl7, Cxcl12, Cd44, Pdpn, Ptgs2, Serpina3n, Sphk1*	10: *Ccl2, Cd14, Hck, Tnfrsf1a, Anxa1, Casp4, Spp1, S1pr3, Thbs1, Tnfrsf26*
3	GO:0043066 negative regulation of apoptotic process	22	5.50 × 10^−7^	2.90 × 10^−4^	10: *Cd44, Timp1, Alox15, Card14, Cdkn1a, Plaur, Rxfp2, Stk40, Stat3, Sphk1*	12: *Bcl3, Clec5a, Hck, Pim1, Tnfrsf1a, Angptl4, Cyr61, Lgals3, Hspb1, Ier3, Socs3, Thbs1*
4	GO:0008284 positive regulation of cell proliferation	21	1.40 × 10^−6^	4.90 × 10^−4^	15: *Cxcl12, Sox11, Timp1, Adm, Alox15, Clcf1, Edn3, Fgf1, Hbegf, Lif, Odc1, Ptgs2, Sfrp2, Stat3, Sphk1*	6: *Hck, Atf3, Lgals3, Il11, Myc, Osmr*
5	GO:0008285 negative regulation of cell proliferation	18	1.50 × 10^−6^	4.90 × 10^−4^	15: *Fosl1, Sox7, Adm, Cdkn1a, Frzb, Hmox1, Inhba, Lif, Ptgs2, Ppp1r15a, Rxfp2, Sfrp2, Stat3, Tesc, Xirp1*	3: *B4galt1, Irf1, Ptges*
6	GO:0034097 response to cytokine	10	2.00 × 10^−6^	5.40 × 10^−4^	5: *Fosl1, Timp1, Ptgs2, Serpina3n, Stat3*	5: *Fos, Osmr, Ptges, Serpine1, Socs3*
7	GO:0014070 response to organic cyclic compound	15	3.20 × 10^−6^	7.20 × 10^−4^	11: *Cd44, Fosl1, Acacb, Angpt2, Cdkn1a, Plin2, Ptgs2, Ppp1r15a, Stat3, Sphk1, Trh*	4: *Fos, Anxa1, Ptges, Socs3*
8	GO:0032496 response to lipopolysaccharide	15	4.50 × 10^−6^	8.90 × 10^−4^	4: *Adm, Pawr, Ptgs2, Serpina3n*	11: *Ccl2, Cd14, Fos, Tnfrsf1a, Csf2rb, Lbp, Ptges, Serpine1, Socs3, Trib1, Tnfrsf26*
9	GO:0043065 positive regulation of apoptotic process	16	8.60 × 10^−6^	1.50 × 10^−3^	10: *Fosl1, Adm, Frzb, Gadd45a, Gadd45g, Hmox1, Pawr, Ptgs2, Ppp1r15a, Sfrp2*	6: *Tnfrsf12a, Anxa1, B4galt1, Cyr61, Tgm2, Tspo*
10	GO:0010628 positive regulation of gene expression	16	2.60 × 10^−5^	4.10 × 10^−3^	10: *Cd44, Sox11, Cyp3a9, Inhba, Lif, Plaur, Pawr, Ppp1r15a, Stat3, Tesc*	6: *Tnfrsf1a, Atf3, Msn, Ret, Serpine1, Vim*
11	GO:0009611 response to wounding	8	3.70 × 10^−5^	5.10 × 10^−3^	2: *Adm, Pawr*	6: *Ccl2, Tnfrsf1a, B4galt1, Gfap, Myc, Zfp36*
12	GO:0042493 response to drug	19	3.90 × 10^−5^	5.10 × 10^−3^	11: *Fosl1, Fosb, S100a10, Acacb, Cdkn1a, Inhba, Lox, Plin2, Ptgs2, Sfrp2, Stat3*	8: *Ccl2, Fos, Anxa1, Myc, Ret, Socs3, Thbs1, Tspo*
13	GO:0032570 response to progesterone	7	6.30 × 10^−5^	7.70 × 10^−3^	3: *Fosl1, Fosb, Sphk1*	4: *Ccl2, Fos, Socs3, Tspo*
14	GO:0045669 positive regulation of osteoblast differentiation	7	1.10 × 10^−4^	1.20 × 10^−2^	4: *Sox11, Gdpd2, Sfrp2, Tmem119*	3: *Cebpd, Clic1, Cyr61*
15	GO:0044344 cellular response to fibroblast growth factor stimulus	6	1.10 × 10^−4^	1.20 × 10^−2^	1: *Cd44*	5: *Ccl2, Myc, Serpine1, Vim, Zfp36*
16	GO:0030335 positive regulation of cell migration	11	1.30 × 10^−4^	1.30 × 10^−2^	6: *Ccl7, Cxcl12, Fgf1, Hbegf, Pdpn, Sphk1*	5: *Fgr, Cyr61, Itga5, Ret, Thbs1*
17	GO:0010811 positive regulation of cell-substrate adhesion	6	1.60 × 10^−4^	1.50 × 10^−2^	2: *Alox15, Fbln2*	4: *Cyr61, Itga5, Spp1, Thbs1*
18	GO:0009612 response to mechanical stimulus	8	1.90 × 10^−4^	1.70 × 10^−2^	4: *Cxcl12, Fosl1, Fosb, Angpt2*	4: *Ccl2, Fos, Mgp, Thbs1*
19	GO:0007565 female pregnancy	8	2.90 × 10^−4^	2.40 × 10^−2^	5: *Fosl1, Fosb, Adm, Cyp3a9, Sphk1*	3: *Fos, Hspb1, Irf1*
20	GO:0071318 cellular response to ATP	4	3.00 × 10^−4^	2.40 × 10^−2^	1: *Ptgs2*	3: *Ccl2, P2ry12, Serpine1*
21	GO:0042127 regulation of cell proliferation	11	3.80 × 10^−4^	2.80 × 10^−2^	2: *Ptgs2, Sfrp2*	9: *Fgr, Hck, S100a11, Tnfrsf1a, Anxa1, B4galt1, Irf1, Serpine1, Tnfrsf26*
22	GO:0033031 positive regulation of neutrophil apoptotic process	3	3.90 × 10^−4^	2.80 × 10^−2^	1: *Cd44*	2: *Anxa1, Hcar2*
23	GO:0032760 positive regulation of tumor necrosis factor biosynthetic process	4	4.90 × 10^−4^	3.40 × 10^−2^	0:	4: *Hspb1, Lbp, Thbs1, Tlr1*
24	GO:0030593 neutrophil chemotaxis	6	5.30 × 10^−4^	3.50 × 10^−2^	2: *Ccl7, Edn3*	4: *Ccl2, Lgals3, Itgam, Spp1*
25	GO:0031100 organ regeneration	7	6.30 × 10^−4^	4.00 × 10^−2^	5: *Cxcl12, Adm, Angpt2, Cdkn1a, Lif*	2: *Ccl2, Socs3*
26	GO:0071222 cellular response to lipopolysaccharide	9	6.90 × 10^−4^	4.20 × 10^−2^	2: *Tnip2, Sbno2*	7: *Ccl2, Cd14, Fcgr2b, Lbp, Serpine1, Tspo, Zfp36*
27	GO:0060252 positive regulation of glial cell proliferation	4	7.40 × 10^−4^	4.40 × 10^−2^	0:	4: *Gfap, Myc, Tspo, Vim*
28	GO:0035690 cellular response to drug	7	8.80 × 10^−4^	4.80 × 10^−2^	3: *Ddc, Ppp1r15a, Rnf149*	4: *Ccl2, Mt2A, Myc, Serpine1*
29	GO:0007155 cell adhesion	11	8.80 × 10^−4^	4.80 × 10^−2^	3: *Cd44, Itgax, Pdpn*	8: *Tnfrsf12a, B4galt1, Cyr61, Gpnmb, Itga5, Itgam, Spp1, Thbs1*
30	GO:0071347 cellular response to interleukin−1	7	9.20 × 10^−4^	4.90 × 10^−2^	3: *Ccl7, Pawr, Serpina3n*	4: *Ccl2, Irf1, Myc, Serpine1*

**Table 3:** Enriched KEGG pathways for all (213) DEGs.

	Term	Count	*p*-Value	MCAO Genes That Did not Respond to LPS	MCAO Genes That Responded to LPS
1	rno04060 Cytokine-cytokine receptor interaction	11	3.70 × 10^−4^	5: *Ccl7, Cxcl12, Clcf1, Il18rap, Lif*	6: *Ccl2, Tnfrsf12a, Tnfrsf1a, Csf2rb, Il11, Osmr*
2	rno05205 Proteoglycans in cancer	11	4.00 × 10^–4^	7: *Cd44, Cdkn1a, Flnc, Hbegf, Plce1, Plaur, Stat3*	4: *Itga5, Msn, Myc, Thbs1*
3	rno04668 TNF signaling pathway	8	7.00 × 10^–4^	3: *Creb3l1, Lif, Ptgs2*	5: *Bcl3, Ccl2, Fos, Tnfrsf1a, Socs3*
4	rno05206 MicroRNAs in cancer	9	7.30 × 10^–4^	4: *Cd44, Cdkn1a, Ptgs2, Stat3*	5: *Pim1, Itga5, Myc, Thbs1, Vim*
5	rno04630 Jak-STAT signaling pathway	8	2.40 × 10^–3^	2: *Lif, Stat3*	6: *Pim1, Csf2rb, Il11, Myc, Osmr, Socs3*
6	rno04066 HIF-1 signaling pathway	7	2.60 × 10^–3^	6: *Pfkfb3, Timp1, Angpt2, Cdkn1a, Hmox1, Stat3*	1: *Serpine1*
7	rno05133 Pertussis	6	3.10× 10^–3^	0:	6: *Cd14, Fos, Itga5, Itgam, Irf1, Serping1*
8	rno04380 Osteoclast differentiation	7	8.30 × 10^–3^	2: Fosl1, Fosb	5: Fos, Fcgr2b, Tnfrsf1a, Ncf4, Socs3
9	rno04010 MAPK signaling pathway	10	8.40 × 10^–3^	5: Fgf1, Flnc, Gadd45a, Gadd45g, Map3k6	5: Cd14, Fos, Tnfrsf1a, Hspb1, Myc
10	rno05152 Tuberculosis	8	1.20 × 10^–2^	2: Itgax, Sphk1	6: Cd14, Fcgr2b, Tnfrsf1a, Itgam, Lbp, Tlr1
11	rno04115 p53 signaling pathway	5	1.50 × 10^–2^	3: Cdkn1a, Gadd45a, Gadd45g	2: Serpine1, Thbs1
12	rno04145 Phagosome	8	1.60 × 10^–2^	0:	8: Cd14, Fcgr2b, Itga5, Itgam, Msr1, Ncf4, Thbs1, Tubb6
13	rno05219 Bladder cancer	4	1.70 × 10^–2^	2: Cdkn1a, Hbegf	2: Myc, Thbs1
14	rno04931 Insulin resistance	6	1.70 × 10^–2^	4: Acacb, Creb3l1, Pygl, Stat3	2: Tnfrsf1a, Socs3
15	rno04640 Hematopoietic cell lineage	5	2.60 × 10^–2^	1: Cd44	4: Cd14, Itga5, Itgam, Il11
16	rno05160 Hepatitis C	6	3.20 × 10^–2^	3: Cdkn1a, Ocln, Stat3	3: Tnfrsf1a, Irf1, Socs3
17	rno04512 ECM-receptor interaction	5	3.30 × 10^–2^	2: Cd44, Col11a2	3: Itga5, Spp1, Thbs1
18	rno04064 NF-kappa B signaling pathway	5	3.30 × 10^–2^	2: Cxcl12, Ptgs2	3: Cd14, Tnfrsf1a, Lbp
19	rno04151 PI3K-Akt signaling pathway	10	4.20 × 10^–2^	5: Angpt2, Creb3l1, Col11a2, Cdkn1a, Fgf1	5: Itga5, Myc, Osmr, Spp1, Thbs1
20	rno04620 Toll-like receptor signaling pathway	5	4.40 × 10^–2^	0:	5: Cd14, Fos, Lbp, Spp1, Tlr1

**Table 4:** Functional annotation clustering of LPS-responded MCAO DEGs.

Category	Term	*n*	*p*-Value	FDR	Genes
Cluster 1 Enrichment Score: 3.63					
GOTERM_BP_DIRECT	Response to wounding	6	3.20 × 10^–5^	4.20 × 10^–3^	*Ccl2, Tnfrsf1a, B4galt1, Gfap, Myc, Zfp36*
GOTERM_BP_DIRECT	Cellular response to fibroblast growth factor stimulus	5	3.90 × 10^–5^	4.50 × 10^–3^	*Ccl2, Myc, Serpine1, Vim, Zfp36*
GOTERM_BP_DIRECT	MAPK cascade	4	9.90 × 10^–3^	2.30 × 10^–1^	*Ccl2, Myc, Ret, Zfp36*
9 record(s) ENSRNOG00000007159 C-C motif chemokine ligand 2 (*Ccl2*) ENSRNOG00000031312 TNF receptor superfamily member 1A (*Tnfrsf1a*) ENSRNOG00000059461 Beta-1,4-galactosyltransferase 1 (*B4galt1*) ENSRNOG00000002919 Glial fibrillary acidic protein (*Gfap*) ENSRNOG00000004500 Myelocytomatosis oncogene (*Myc*) ENSRNOG00000014751 Ret proto-oncogene (*Ret*) ENSRNOG00000001414 Serpin family E member 1 (*Serpine1*) ENSRNOG00000018087 Vimentin (*Vim*) ENSRNOG00000058388 Zinc finger protein 36 (*Zfp36*)					
Cluster 2 Enrichment Score: 3.39					
UP_KEYWORDS	Innate immunity	8	4.90 × 10^–7^	3.50 × 10^–5^	*Cd14, Fgr, Hck, Anxa1, Lgals3, Irf1, Lbp, Serping1*
UP_KEYWORDS	Immunity	8	2.40 × 10^–5^	8.80 × 10^–4^	*Cd14, Fgr, Hck, Anxa1, Lgals3, Irf1, Lbp, Serping1*
GOTERM_BP_DIRECT	Innate immune response	9	2.50 × 10^–5^	4.20 × 10^–3^	*Clec5a, Cd14, Fgr, Hck, Anxa1, Lgals3, Lbp, Ptx3, Serping1*
GOTERM_BP_DIRECT	Defense response to Gram-positive bacterium	3	6.60 × 10^–2^	6.20 × 10^–1^	*Fgr, Hck, Lbp*
UP_KEYWORDS	Lipoprotein	3	5.60 × 10^–1^	9.50 × 10^–1^	*Cd14, Fgr, Hck*
10 record(s) ENSRNOG00000026306 C-type lectin domain family 5, member A (*Clec5a*) ENSRNOG00000017819 CD14 molecule (*Cd14*) ENSRNOG00000009912 FGR proto-oncogene, Src family tyrosine kinase (*Fgr*) ENSRNOG00000009331 HCK proto-oncogene, Src family tyrosine kinase (*Hck*) ENSRNOG00000017469 Annexin A1 (*Anxa1*) ENSRNOG00000010645 Galectin 3 (*Lgals3*) ENSRNOG00000008144 Interferon regulatory factor 1 (*Irf1*) ENSRNOG00000014532 Lipopolysaccharide binding protein (*Lbp*) ENSRNOG00000012280 Pentraxin 3 (*Ptx3*) ENSRNOG00000007457 Serpin family G member 1 (*Serping1*)					

## Supplementary Materials

The following are available online at https://www.mdpi.com/article/10.3390/biomedicines9121840/s1, Table S1: List of all differentially expressed genes in the hippocampus of MCAO vs. SHAM at 24 h; Table S2–S7: Functional annotation.
