# Supplementary material for: Identifying the Involvement of Pro-Inflammatory Signal in Hippocampal Gene Expression Changes after Experimental Ischemia: Transcriptome-Wide Analysis
Source: Biomedicines. 2021 Dec 5;9(12):1840. doi: 10.3390/biomedicines9121840 (PMC8698395; doi:10.3390/biomedicines9121840)
Supplement: Supplementary file 1 [file biomedicines-09-01840-s001.zip › biomedicines-1487492-SI.pdf]

Table S1. List of differentially expressed genes with padj < 0.05 and |log2FC|> 0.95
in the hippocampus of MCAO vs. SHAM at 24 h.

	Ensembl gene ID	Gene symbol	log2FC	pvalue	padj
1	ENSRNOG00000010478	Serpina3n	10,1923	4,70E-06	6,00E-04
2	ENSRNOG00000000239	Ccl7	7,9959	5,90E-10	2,40E-07
3	ENSRNOG00000011824	Trh	6,4779	5,80E-06	7,10E-04
4	ENSRNOG00000007159	Ccl2	6,3151	1,50E-13	1,20E-10
5	ENSRNOG00000043451	Spp1	6,1084	5,10E-10	2,20E-07
6	ENSRNOG00000002918	FAM187A	5,6204	5,40E-10	2,30E-07
7	ENSRNOG00000001414	Serpine1	4,9364	8,50E-08	2,00E-05
8	ENSRNOG00000007002	Lif	4,9064	3,90E-14	4,20E-11
9	ENSRNOG00000011250	Inmt - CC	4,6059	5,50E-06	6,90E-04
10	ENSRNOG00000023546	Hspb1	4,5476	6,90E-06	8,50E-04
11	ENSRNOG00000010208	Timp1	4,3324	2,40E-08	6,40E-06
12	ENSRNOG00000002946	Socs3	4,2989	4,40E-24	1,60E-20
13	ENSRNOG00000013588	Glra1	3,9594	2,60E-04	1,50E-02
14	ENSRNOG00000002905	Ccdc103	3,9383	5,00E-11	2,70E-08
15	ENSRNOG00000012067	Fam111a	3,9220	1,40E-05	1,50E-03
16	ENSRNOG00000017819	Cd14	3,8398	5,50E-18	1,10E-14
17	ENSRNOG00000000187	Csf2rb	3,6937	8,20E-33	1,20E-28
18	ENSRNOG00000037931	Plaur	3,5176	1,20E-03	4,40E-02
19	ENSRNOG00000034174	Bpifb4	3,4861	7,30E-04	3,00E-02
20	ENSRNOG00000012843	Aspg	3,4842	4,50E-11	2,50E-08
21	ENSRNOG00000017410	Loxhd1	3,4435	9,80E-08	2,20E-05
22	ENSRNOG00000012280	Ptx3	3,4406	9,80E-06	1,10E-03
23	ENSRNOG00000011316	Fam167a	3,3891	4,60E-11	2,50E-08
24	ENSRNOG00000008412	Gprc5a	3,2742	2,10E-05	2,00E-03
25	ENSRNOG00000003745	Atf3	3,2549	1,70E-12	1,20E-09
26	ENSRNOG00000017386	Il11	3,0719	1,20E-05	1,40E-03
27	ENSRNOG00000010645	Lgals3	3,0697	5,60E-08	1,40E-05
28	ENSRNOG00000043416	Bcl3	3,0608	1,10E-13	9,70E-11
29	ENSRNOG00000014838	Glipr2	3,0438	2,60E-09	8,90E-07
30	ENSRNOG00000036703	Itgax	2,9950	1,60E-05	1,60E-03
31	ENSRNOG00000014532	Lbp	2,9507	2,80E-04	1,50E-02
32	ENSRNOG00000014426	Lox	2,9115	5,80E-11	2,90E-08
33	ENSRNOG00000012886	Maff	2,9057	1,50E-22	4,30E-19
34	ENSRNOG00000013090	Gadd45g	2,8427	2,50E-06	3,60E-04
35	ENSRNOG00000012779	Msr1	2,8403	2,00E-04	1,20E-02
36	ENSRNOG00000003546	Tnfrsf12a	2,7672	1,30E-09	4,90E-07
37	ENSRNOG00000005037	Kif18a	2,7014	4,10E-07	7,60E-05
38	ENSRNOG00000002919	Gfap	2,6165	3,50E-07	6,70E-05
39	ENSRNOG00000006094	Cd44	2,6080	8,30E-06	1,00E-03
40	ENSRNOG00000026306	Clec5a	2,5953	6,10E-05	4,40E-03
41	ENSRNOG00000000897	Rxfp2	2,5627	5,60E-05	4,20E-03
42	ENSRNOG00000014117	Hmox1	2,4746	1,50E-09	5,50E-07
43	ENSRNOG00000014320	Inhba	2,4568	2,60E-07	5,30E-05
44	ENSRNOG00000018752	Clcf1	2,4374	3,80E-08	1,00E-05
45	ENSRNOG00000006320	Ptges	2,4369	2,90E-07	5,70E-05
46	ENSRNOG00000008015	Fos	2,4209	3,50E-27	2,50E-23
47	ENSRNOG00000010513	Tfpi2	2,3926	2,50E-07	5,30E-05
48	ENSRNOG00000012881	Fgl2	2,3808	1,30E-16	1,90E-13
49	ENSRNOG00000010105	S100a11	2,3801	1,50E-05	1,50E-03
50	ENSRNOG00000033192	Osmr	2,3718	5,40E-17	8,50E-14
51	ENSRNOG00000062141	Slc18a3	2,3669	5,80E-05	4,30E-03
52	ENSRNOG00000008586	Aldh1l2	2,3581	8,10E-04	3,30E-02
53	ENSRNOG00000058568	Dhrs9	2,3101	3,20E-06	4,20E-04
54	ENSRNOG00000054218	Il18rap	2,2855	2,70E-04	1,50E-02
55	ENSRNOG00000018371	Tubb6	2,2144	4,90E-04	2,30E-02
56	ENSRNOG00000014524	S1pr3	2,2052	9,10E-11	4,30E-08
57	ENSRNOG00000010626	Sphk1	2,1790	3,30E-04	1,70E-02
58	ENSRNOG00000012892	Abca4	2,1535	4,80E-06	6,10E-04
59	ENSRNOG00000001607	Adamts1	2,1521	8,70E-21	2,10E-17

60	ENSRNOG00000010549	Tspo	2,1508	1,90E-05	1,90E-03
61	ENSRNOG00000006940	Ncf4	2,1310	2,70E-06	3,70E-04
62	ENSRNOG00000005615	Gadd45a	2,1232	1,60E-08	5,00E-06
63	ENSRNOG00000008215	Trim47	2,1075	7,60E-14	7,20E-11
64	ENSRNOG00000058388	Zfp36	2,1075	3,70E-26	1,80E-22
65	ENSRNOG00000012049	Sox7	1,9909	5,40E-05	4,10E-03
66	ENSRNOG00000011503	Shb	1,9502	1,80E-06	2,70E-04
67	ENSRNOG00000046452	Fcgr2b	1,9363	6,10E-09	2,00E-06
68	ENSRNOG00000004273	Ifitm1	1,8606	3,10E-05	2,70E-03
69	ENSRNOG00000047363	Kcnk13	1,8458	7,10E-05	5,00E-03
70	ENSRNOG00000000878	Slc44a4	1,8455	8,00E-04	3,20E-02
71	ENSRNOG00000002525	Ptgs2	1,8450	1,40E-03	4,90E-02
72	ENSRNOG00000015113	Mocos	1,8350	2,20E-05	2,10E-03
73	ENSRNOG00000000521	Cdkn1a	1,8179	1,20E-08	3,60E-06
74	ENSRNOG00000000529	Pim1	1,8163	3,90E-08	1,00E-05
75	ENSRNOG00000043098	Mt2A	1,8050	9,20E-09	2,90E-06
76	ENSRNOG00000038722	Tlr1	1,7673	4,70E-04	2,30E-02
77	ENSRNOG00000050869	Cebpd	1,7634	2,50E-17	4,50E-14
78	ENSRNOG00000004100	Trib1	1,7581	1,80E-09	6,40E-07
79	ENSRNOG00000027030	Adm	1,7415	4,90E-07	8,90E-05
80	ENSRNOG00000016678	Angptl2	1,7340	1,70E-04	1,10E-02
81	ENSRNOG00000043486	Tnfrsf26	1,7262	5,50E-05	4,10E-03
82	ENSRNOG00000008816	Gpnmb	1,6980	1,90E-08	5,50E-06
83	ENSRNOG00000018087	Vim	1,6966	1,10E-03	3,90E-02
84	ENSRNOG00000020298	Bag3	1,6777	2,90E-07	5,70E-05
85	ENSRNOG00000004500	Myc	1,6698	4,10E-12	2,80E-09
86	ENSRNOG00000027784	Tsku	1,6681	1,20E-04	7,50E-03
87	ENSRNOG00000005935	A3galt2	1,6636	4,70E-07	8,60E-05
88	ENSRNOG00000061379	C7	1,6624	2,70E-06	3,80E-04
89	ENSRNOG00000008676	Emp1	1,6386	5,90E-08	1,50E-05
90	ENSRNOG00000039496	Plp2	1,6358	1,00E-04	6,90E-03
91	ENSRNOG00000037085	Xirp1	1,6347	3,40E-04	1,80E-02
92	ENSRNOG00000014961	Pdpn	1,5937	8,30E-07	1,40E-04
93	ENSRNOG00000012865	Parp3	1,5842	3,00E-05	2,60E-03
94	ENSRNOG00000002434	Tmem100	1,5803	1,20E-15	1,60E-12
95	ENSRNOG00000017469	Anxa1	1,5545	2,80E-05	2,50E-03
96	ENSRNOG00000019141	Ch25h	1,5412	7,10E-07	1,20E-04
97	ENSRNOG00000013668	Capg	1,5287	1,40E-03	4,90E-02
98	ENSRNOG00000019202	Pvr	1,5258	6,60E-05	4,70E-03
99	ENSRNOG00000011647	S100a6	1,5162	1,60E-04	9,90E-03
100	ENSRNOG00000036677	Slc16a3	1,4975	2,60E-05	2,30E-03
101	ENSRNOG00000036834	Gpr84	1,4884	2,20E-07	4,70E-05
102	ENSRNOG00000045829	Thbs1	1,4860	1,50E-06	2,40E-04
103	ENSRNOG00000007281	Flnc	1,4670	5,30E-12	3,40E-09
104	ENSRNOG00000006828	Baz1a	1,4668	3,20E-06	4,30E-04
105	ENSRNOG00000057153	Pla1a	1,4577	2,50E-07	5,30E-05
106	ENSRNOG00000015160	Gem	1,4530	2,60E-09	8,90E-07
107	ENSRNOG00000008936	Map3k6	1,4518	2,70E-05	2,40E-03
108	ENSRNOG00000031312	Tnfrsf1a	1,4507	6,80E-14	6,90E-11
109	ENSRNOG00000010617	Scube1	1,4478	1,80E-05	1,80E-03
110	ENSRNOG00000019780	Sypl2	1,4430	2,50E-05	2,30E-03
111	ENSRNOG00000027024	Rgs16	1,4375	5,60E-12	3,50E-09
112	ENSRNOG00000016413	Pstpip1	1,4343	1,10E-04	7,40E-03
113	ENSRNOG00000030034	Sox11	1,4303	3,20E-04	1,70E-02
114	ENSRNOG00000023226	S100a10	1,4269	2,40E-04	1,40E-02
115	ENSRNOG00000009912	Fgr	1,4260	3,40E-05	2,90E-03
116	ENSRNOG00000014751	Ret	1,4078	2,20E-08	6,10E-06
117	ENSRNOG00000053787	Mdfic	1,3975	1,40E-05	1,50E-03
118	ENSRNOG00000047367	Card14	1,3881	9,20E-04	3,50E-02
119	ENSRNOG00000036682	Pycr1	1,3876	3,20E-04	1,70E-02
120	ENSRNOG00000033697	Casp4	1,3679	8,70E-06	1,00E-03
121	ENSRNOG00000019742	Stat3	1,3679	3,50E-14	4,10E-11

122	ENSRNOG00000057451	Itga5	1,3618	4,50E-05	3,60E-03
123	ENSRNOG00000003888	Rgs13	1,3526	4,80E-04	2,30E-02
124	ENSRNOG00000018646	Hbegf	1,3463	4,50E-05	3,70E-03
125	ENSRNOG00000019728	Itgam	1,3416	3,80E-05	3,10E-03
126	ENSRNOG00000007338	Fbln2	1,3402	4,80E-05	3,70E-03
127	ENSRNOG00000016696	Angpt2	1,3323	1,60E-04	9,70E-03
128	ENSRNOG00000013987	Sbno2	1,3188	5,00E-10	2,20E-07
129	ENSRNOG00000029682	Clic1	1,2976	1,60E-06	2,40E-04
130	ENSRNOG00000007545	Angptl4	1,2853	6,90E-07	1,20E-04
131	ENSRNOG00000026653	Hcar2 (Niacr1)	1,2754	1,30E-03	4,50E-02
132	ENSRNOG00000007457	Serping1	1,2730	7,60E-05	5,30E-03
133	ENSRNOG00000009450	Hcn4	1,2723	2,70E-04	1,50E-02
134	ENSRNOG00000016687	Ssc5d	1,2686	3,60E-04	1,90E-02
135	ENSRNOG00000000827	Ier3	1,2621	3,20E-09	1,10E-06
136	ENSRNOG00000050794	Pdlim4	1,2571	3,20E-07	6,30E-05
137	ENSRNOG00000013946	Rnf149	1,2520	9,20E-05	6,20E-03
138	ENSRNOG00000019810	Des	1,2429	3,10E-04	1,70E-02
139	ENSRNOG00000011913	Cp	1,2422	3,80E-07	7,10E-05
140	ENSRNOG00000014258	Rab32	1,2334	2,20E-04	1,30E-02
141	ENSRNOG00000005214	Plek	1,2324	2,20E-04	1,30E-02
142	ENSRNOG00000005695	Mgp	1,2155	8,30E-13	6,30E-10
143	ENSRNOG00000019447	Ecel1	1,2072	2,20E-04	1,20E-02
144	ENSRNOG00000043113	Fam43b	1,1976	1,10E-03	4,20E-02
145	ENSRNOG00000008301	Tagln2	1,1975	2,80E-07	5,70E-05
146	ENSRNOG00000025679	Stk40	1,1968	9,40E-07	1,50E-04
147	ENSRNOG00000012956	Tgm2	1,1898	1,30E-05	1,40E-03
148	ENSRNOG00000004972	Upp1	1,1874	1,70E-05	1,70E-03
149	ENSRNOG00000014350	Ccn1 (Cyr61)	1,1810	8,60E-04	3,30E-02
150	ENSRNOG00000005917	Pawr	1,1739	1,50E-05	1,50E-03
151	ENSRNOG00000015382	Arid5a	1,1705	3,60E-13	2,90E-10
152	ENSRNOG00000025476	Tmem252	1,1695	8,40E-05	5,80E-03
153	ENSRNOG00000007060	Plin2	1,1524	1,20E-03	4,40E-02
154	ENSRNOG00000015948	Slc1a5	1,1348	5,30E-04	2,40E-02
155	ENSRNOG00000005248	Slc1a4	1,1334	2,90E-11	1,80E-08
156	ENSRNOG00000023013	Marchf3	1,1237	1,50E-04	9,10E-03
157	ENSRNOG00000056457	Gpd1	1,1201	1,30E-09	4,90E-07
158	ENSRNOG00000014647	Cbfb	1,1161	5,00E-04	2,30E-02
159	ENSRNOG00000046667	Fosb	1,1137	9,20E-07	1,50E-04
160	ENSRNOG00000006388	Pygl	1,1109	7,80E-04	3,20E-02
161	ENSRNOG00000001128	Tesc	1,0935	1,20E-03	4,30E-02
162	ENSRNOG00000003219	Trim16	1,0842	1,00E-05	1,20E-03
163	ENSRNOG00000016166	Pdlim1	1,0719	4,20E-05	3,50E-03
164	ENSRNOG00000008492	Cfap45	1,0576	6,10E-04	2,70E-02
165	ENSRNOG00000030118	Msn	1,0504	1,70E-04	1,00E-02
166	ENSRNOG00000013867	Fgf1	1,0491	3,10E-04	1,70E-02
167	ENSRNOG00000010362	Anxa2	1,0486	1,20E-03	4,50E-02
168	ENSRNOG00000049714	Asap3	1,0459	2,90E-05	2,50E-03
169	ENSRNOG00000014276	Plce1	1,0391	4,50E-04	2,20E-02
170	ENSRNOG00000033433	Csrnp1	1,0325	4,20E-05	3,50E-03
171	ENSRNOG00000056786	Piezo1	1,0277	1,50E-05	1,50E-03
172	ENSRNOG00000005413	Creb3l1	1,0221	3,70E-06	4,80E-04
173	ENSRNOG00000020552	Fosl1	1,0140	6,60E-05	4,70E-03
174	ENSRNOG00000016491	Fam110d	1,0132	8,70E-05	5,90E-03
175	ENSRNOG00000013805	Tnip2	1,0030	1,80E-04	1,10E-02
176	ENSRNOG00000008144	Irf1	0,9875	8,00E-05	5,50E-03
177	ENSRNOG00000009331	Hck	0,9868	1,80E-04	1,10E-02
178	ENSRNOG00000018911	Pfkfb3	0,9846	1,30E-09	4,90E-07
179	ENSRNOG00000012546	Frmpd1	0,9578	6,30E-07	1,10E-04
180	ENSRNOG00000020938	Ppp1r15a	0,9563	5,00E-05	3,90E-03
181	ENSRNOG00000059461	B4galt1	0,9561	2,10E-04	1,20E-02
182	ENSRNOG00000005424	Odc1	0,9520	7,30E-05	5,20E-03
183	ENSRNOG00000018297	Ocln	-0,9501	3,20E-06	4,20E-04

184	ENSRNOG00000007765	Frzb	-0,9501	2,10E-05	2,00E-03
185	ENSRNOG00000016494	Tmem204	-0,9515	1,60E-05	1,60E-03
186	ENSRNOG00000039759	Gpr34	-0,9859	1,00E-07	2,30E-05
187	ENSRNOG00000021027	Dbp	-1,0006	8,20E-04	3,30E-02
188	ENSRNOG00000028908	Eppin	-1,0079	1,10E-03	4,10E-02
189	ENSRNOG00000000463	Col11a2	-1,0488	4,60E-05	3,70E-03
190	ENSRNOG00000007044	L3mbtl1	-1,0679	5,10E-04	2,40E-02
191	ENSRNOG00000013902	P2ry12	-1,0722	5,10E-06	6,40E-04
192	ENSRNOG00000009465	Sfrp2	-1,0930	1,80E-04	1,10E-02
193	ENSRNOG00000027730	Nxpe1	-1,1010	4,00E-04	2,00E-02
194	ENSRNOG00000028781	Abcc6	-1,1054	2,00E-05	1,90E-03
195	ENSRNOG00000003237	Tp53bp2	-1,1056	4,70E-05	3,70E-03
196	ENSRNOG00000027767	Slc38a5	-1,1060	6,90E-04	2,90E-02
197	ENSRNOG00000000658	Acacb	-1,1102	1,60E-04	9,90E-03
198	ENSRNOG00000007102	Acss1	-1,1120	3,10E-06	4,20E-04
199	ENSRNOG00000003098	Prom1	-1,1295	1,90E-05	1,90E-03
200	ENSRNOG00000046643	Cyp3a9	-1,1374	1,40E-03	5,00E-02
201	ENSRNOG00000022910	Emcn	-1,1520	2,60E-06	3,70E-04
202	ENSRNOG00000004786	Cyp4f1	-1,1528	8,10E-04	3,30E-02
203	ENSRNOG00000007837	Acot11	-1,1532	5,30E-05	4,00E-03
204	ENSRNOG00000018131	Slc16a4	-1,1546	8,50E-04	3,30E-02
205	ENSRNOG00000003120	Prelp	-1,1742	8,30E-08	2,00E-05
206	ENSRNOG00000002800	Gdpd2	-1,2062	2,50E-05	2,30E-03
207	ENSRNOG00000004327	Ddc	-1,2603	4,40E-04	2,20E-02
208	ENSRNOG00000007477	Edn3	-1,2810	1,40E-04	8,90E-03
209	ENSRNOG00000002365	Itm2a	-1,3899	6,30E-08	1,50E-05
210	ENSRNOG00000000700	Tmem119	-1,4331	5,00E-04	2,30E-02
211	ENSRNOG00000013589	Cxcl12	-1,5183	1,90E-08	5,60E-06
212	ENSRNOG00000019183	Alox15	-2,1047	1,20E-05	1,30E-03
213	ENSRNOG00000016164	Fcrl2	-2,3478	2,50E-05	2,30E-03

Table S2a. Enriched GO biological process terms for commonly responding to MCAO and LPS (84) MCAO DEGs

	Term	N	P-value	FDR	Genes
1	GO:0032496 response to lipopolysaccharide	11	7,1E-7	6,5E-4	Ccl2, Cd14, Fos, Tnfrsf1a, Csf2rb, Lbp, Ptges, Serpine1, Socs3, Trib1, Tnfrsf26
2	GO:0006954 inflammatory response	10	1,0E-5	4,2E-3	Ccl2, Cd14, Hck, Tnfrsf1a, Anxa1, Casp4, Spp1, S1pr3, Thbs1, Tnfrsf26
3	GO:0042127 regulation of cell proliferation	9	1,4E-5	4,2E-3	Fgr, Hck, S100a11, Tnfrsf1a, Anxa1, B4galt1, Irf1, Serpine1, Tnfrsf26
4	GO:0043066 negative regulation of apoptotic process	12	2,8E-5	4,7E-3	Bcl3, Clec5a, Hck, Pim1, Tnfrsf1a, Angptl4, Cyr61, Lgals3, Hspb1, Ier3, Socs3, Thbs1
5	GO:0045087 innate immune response	9	3,0E-5	4,7E-3	Clec5a, Cd14, Fgr, Hck, Anxa1, Lgals3, Lbp, Ptx3, Serping1
6	GO:0032760 positive regulation of tumor necrosis factor biosynthetic process	4	3,5E-5	4,7E-3	Hspb1, Lbp, Thbs1, Tlr1
7	GO:0009611 response to wounding	6	3,6E-5	4,7E-3	Ccl2, Tnfrsf1a, B4galt1, Gfap, Myc, Zfp36
8	GO:0044344 cellular response to fibroblast growth factor stimulus	5	4,3E-5	5,0E-3	Ccl2, Myc, Serpine1, Vim, Zfp36
9	GO:0060252 positive regulation of glial cell proliferation	4	5,3E-5	5,4E-3	Gfap, Myc, Tspo, Vim
10	GO:0071222 cellular response to lipopolysaccharide	7	1,3E-4	1,2E-2	Ccl2, Cd14, Fcgr2b, Lbp, Serpine1, Tspo, Zfp36
11	GO:0007155 cell adhesion	8	2,2E-4	1,7E-2	Tnfrsf12a, B4galt1, Cyr61, Gpnmb, Itga5, Itgam, Spp1, Thbs1
12	GO:0045766 positive regulation of angiogenesis	6	2,2E-4	1,7E-2	Tnfrsf1a, Cyr61, Lgals3, Hspb1, Serpine1, Thbs1
13	GO:0007568 aging	8	6,3E-4	4,4E-2	Ccl2, Fos, Cp, Hspb1, Serping1, Socs3, Tspo, Vim
14	GO:0007229 integrin-mediated signaling pathway	5	7,0E-4	4,8E-2	Adamts1, Fgr, Itga5, Itgam, Plek

Table S2b. Enriched GO biological process terms for not responding to LPS (129) MCAO DEGs

	Term	N	P-value	FDR	Genes
1	GO:0008285 negative regulation of cell proliferation	15	2,2E-7	2,2E-4	Fosl1, Sox7, Adm, Cdkn1a, Frzb*, Hmox1, Inhba, Lif, Ptgs2, Ppp1r15a, Rxfp2, Sfrp2*, Stat3, Tesc, Xirp1
2	GO:0008284 positive regulation of cell proliferation	15	7,3E-6	3,7E-3	Cxcl12*, Sox11, Timp1, Adm, Alox15*, Clcf1, Edn3*, Fgf1, Hbegf, Lif, Odc1, Ptgs2, Sfrp2*, Stat3, Sphk1
3	GO:0014070 response to organic cyclic compound	11	1,5E-5	5,2E-3	Cd44, Fosl1, Acacb*, Angpt2, Cdkn1a, Plin2, Ptgs2, Ppp1r15a, Stat3, Sphk1, Trh

Table S3a. Enriched GO molecular function terms for all (213) MCAO DEGs

	Term	N	P-value	FDR	MCAO genes that did not respond to LPS	MCAO genes that responded to LPS
1	GO:0008201 heparin binding	10	4,4E-5	1,7E-2	4: Ccl7, Fgf1, Hbegf, Prelp	6: Adamts1, Ccl2, Cyr61, Gpnmb, Itgam, Thbs1
2	GO:0050840 extracellular matrix binding	5	2,3E-4	4,5E-2	2: Fbln2, Ssc5d	3: Cyr61, Spp1, Thbs1
3	GO:0044548 S100 protein binding	4	3,8E-4	4,9E-2	2: S100a6, Fgf1	2: S100a11, Anxa2

Table S3b. Enriched GO molecular function terms for commonly responding to MCAO and LPS (84) MCAO DEGs

	Term	N	P-value	FDR	Genes
1	GO:0005178 integrin binding	6	1,1E-4	2,3E-2	Cyr61, Gfap, Gpnmb, Itga5, S1pr3, Thbs1

Table S4a. Enriched GO cellular component terms for all (213) MCAO DEGs

	Term	N	P-value	FDR	MCAO genes that did not respond to LPS	MCAO genes that responded to LPS
1	GO:0005615 extracellular space	43	2,2E-10	5,0E-8	20: Ccl7, Cxcl12, Timp1, Adm, Angpt2, Edn3, Eppin,	23: Ccl2, Cd14, S100a11, Tnfrsf1a, Angptl4, Anxa1,

					Fgf1, Frzb, Hbegf, Inhba, Lif, Lox, Prelp, Prom1, Ssc5d, Sfrp2, Serpina3n, Scube1, Tsku	Anxa2, B4galt1, Cp, Clic1, Lgals3, Hspb1, Itga5, Itgam, Il11, Lbp, Mgp, Msn, Ptx3, Spp1, Serpine1, Serping1, Thbs1
2	GO:0009986 cell surface	23	9,9E-7	1,1E-4	8: Cd44, Eppin, Hbegf, Itgax, PVR, Prom1, Scube1, Slc1a4	15: Clec5a, Cd14, Fcgr2b, Tnfrsf12a, Tnfrsf1a, Anxa1, Anxa2, B4galt1, Lgals3, Itga5, Itgam, Lbp, Msn, P2ry12, Thbs1
3	GO:0009897 external side of plasma membrane	15	1,5E-6	1,1E-4	6: Cxcl12, Cd44, Glra1, Itgax, Pdpn, Scube1	9: Cd14, Fcgr2b, B4galt1, Lgals3, Itga5, Itgam, Osmr, P2ry12, Thbs1
4	GO:0031012 extracellular matrix	14	4,5E-6	2,5E-4	4: Timp1, Fbln2, Prelp, Ssc5d	10: Adamts1, Cyr61, Lgals3, Hspb1, Mgp, Serpine1, Thbs1, Tfpi2, Tgm2, Vim
5	GO:0070062 extracellular exosome	54	5,4E-6	2,5E-4	24: Cxcl12, Cd44, Frmpd1, Gprc5a, S100a10, S100a6, Timp1, Acot11, Aldh1l2, Angptl2, Clcf1, C7, Ddc, Fbln2, Gpd1, Itm2a, Pygl, Plaur, PVR, Prelp, Prom1, Plp2, Rnf149, Slc1a4	30: Cd14, Fgr, Glipr2, S100a11, Anxa1, Anxa2, B4galt1, Capg, Cp, Clic1, Des, Fgl2, Lgals3, Hspb1, Itgam, Lbp, Mgp, Msn, Pla1a, Spp1, Serpine1, Serping1, Slc1a5, Slc44a4, Thbs1, Tagln2, Tgm2, Tspo, Tubb6, Vim
6	GO:0001726 ruffle	8	5,0E-5	1,9E-3	5: Asap3, S100a6, Kif18a, Pdpn, Tesc	3: Tnfrsf12a, Anxa2,
7	GO:0005576 extracellular region	20	3,2E-4	1,0E-2	14: Bpifb4, Timp1, Clcf1, Edn3, Eppin, Fgf1, Frzb, Inhba, Lif, Plaur, Sfrp2, Serpina3n, Trh, Tsku	6: Ccl2, Angptl4, B4galt1, Cyr61, Serpine1, Slc1a5
8	GO:0005925 focal adhesion	13	1,4E-3	4,0E-2	6: Asap3, Cd44, Pdlim1, Flnc, Plaur, PVR,	7: Hck, Anxa1, Hspb1, Itga5, Msn, Tgm2, Vim
9	GO:0048471 perinuclear region of cytoplasm	17	1,9E-3	4,5E-2	8: S100a6, Cdkn1a, Hmox1, Hcn4, Inhba, Odc1, Tmem100, Tp53bp2	9: Ccl2, Anxa2, Clic1, Mt2A, Msn, Myc, Ptges, Spp1, Vim
10	GO:0031982 vesicle	8	2,0E-3	4,5E-2	1: Prom1	7: A3galt2, Anxa1, Anxa2, Clic1, Msn, Spp1, Tagln2

Table S4b. Enriched GO cellular component terms for commonly responding to MCAO and LPS (84) MCAO DEGs

	Term	N	P-value	FDR	Genes
1	GO:0005615 extracellular space	23	4,7E-8	8,4E-6	Ccl2, Cd14, S100a11, Tnfrsf1a, Angptl4, Anxa1, Anxa2, B4galt1, Cp, Clic1, Lgals3, Hspb1, Itga5, Itgam, Il11, Lbp, Mgp, Msn, Ptx3, Spp1, Serpine1, Serping1, Thbs1
2	GO:0009986 cell surface	15	5,2E-7	4,6E-5	Clec5a, Cd14, Fcgr2b, Tnfrsf12a, Tnfrsf1a, Anxa1, Anxa2, B4galt1, Lgals3, Itga5, Itgam, Lbp, Msn, P2ry12, Thbs1
3	GO:0070062 extracellular exosome	30	1,9E-6	9,0E-5	Cd14, Fgr, Glipr2, S100a11, Anxa1, Anxa2, B4galt1, Capg, Cp, Clic1, Des, Fgl2, Lgals3, Hspb1,<br>Itgam, Lbp, Mgp, Msn, Pla1a, Spp1, Serpine1, Serping1, Slc1a5, Slc44a4, Thbs1, Tagln2, Tgm2, Tspo, Tubb6, Vim
4	GO:0031012 extracellular matrix	10	2,0E-6	9,0E-5	Adamts1, Cyr61, Lgals3, Hspb1, Mgp, Serpine1, Thbs1, Tfpi2, Tgm2, Vim
5	GO:0009897 external side of plasma membrane	9	2,6E-5	9,3E-4	Cd14, Fcgr2b, B4galt1, Lgals3, Itga5, Itgam, Osmr, P2ry12, Thbs1
6	GO:0031982 vesicle	7	8,5E-5	2,5E-3	A3galt2, Anxa1, Anxa2, Clic1, Msn, Spp1, Tagln2
7	GO:0072562 blood microparticle	5	1,9E-3	4,7E-2	Angptl4, Cp, Clic1, Msn, Serping1

Table S4c. Enriched GO cellular component terms for not responding to LPS (129) MCAO DEGs

	Term	N	P-value	FDR	Genes
1	GO:0005576 extracellular region	14	6,3E-4	4,3E-2	Bpifb4, Timp1, Clcf1, Edn3, Eppin, Fgf1, Frzb, Inhba, Lif, Plaur, Sfrp2, Serpina3n, Trh, Tsku
2	GO:0005615 extracellular space	20	6,4E-4	4,3E-2	Ccl7, Cxcl12, Timp1, Adm, Angpt2, Edn3, Eppin, Fgf1, Frzb, Hbegf, Inhba, Lif, Lox, Prelp, Prom1, Ssc5d, Sfrp2, Serpina3n, Scube1, Tsku

Table S5a. Enriched KEGG pathways for commonly responding to MCAO and LPS (84) MCAO DEGs

	Term	N	P-value	Genes
1	rno05133 Pertussis	6	1,2E-4	Cd14, Fos, Itga5, Itgam, Irf1, Serping1
2	rno04145 Phagosome	8	2,8E-4	Cd14, Fcgr2b, Itga5, Itgam, Msr1, Ncf4, Thbs1, Tubb6
3	rno04630 Jak-STAT signaling pathway	6	2,0E-3	Pim1, Csf2rb, Il11, Myc, Osmr, Socs3
4	rno04620 Toll-like receptor signaling pathway	5	3,9E-3	Cd14, Fos, Lbp, Spp1, Tlr1
5	rno04668 TNF signaling pathway	5	6,0E-3	Bcl3, Ccl2, Fos, Tnfrsf1a, Socs3
6	rno05152 Tuberculosis	6	7,2E-3	Cd14, Fcgr2b, Tnfrsf1a, Itgam, Lbp, Tlr1
7	rno05230 Central carbon metabolism in cancer	4	8,9E-3	Myc, Ret, Slc1a5, Slc16a3
8	rno04060 Cytokine-cytokine receptor interaction	6	1,0E-2	Ccl2, Tnfrsf12a, Tnfrsf1a, Csf2rb, Il11, Osmr
9	rno04380 Osteoclast differentiation	5	1,1E-2	Fos, Fcgr2b, Tnfrsf1a, Ncf4, Socs3
10	rno05206 MicroRNAs in cancer	5	1,5E-2	Pim1, Itga5, Myc, Thbs1, Vim
11	rno04640 Hematopoietic cell lineage	4	1,7E-2	Cd14, Itga5, Itgam, Il11
12	rno05142 Chagas disease (American trypanosomiasis)	4	3,5E-2	Ccl2, Fos, Tnfrsf1a, Serpine1
13	rno05166 HTLV-I infection	6	4,3E-2	Fos, Tnfrsf1a, Atf3, Myc, Tspo, Zfp36

Table S5b. Enriched KEGG pathways for not responding to LPS (129) MCAO DEGs

	Term	N	P-value	Genes
1	rno04066 HIF-1 signaling pathway	6	6,2E-4	Pfkfb3, Timp1, Angpt2, Cdkn1a, Hmox1, Stat3
2	rno05205 Proteoglycans in cancer	7	2,4E-3	Cd44, Cdkn1a, Flnc, Hbegf, Plce1, Plaur, Stat3
3	rno04931 Insulin resistance	4	3,9E-2	Acacb, Creb3l1, Pygl, Stat3
4	Cocaine addiction	3	3,9E-2	Fosb, Creb3l1, Ddc
5	Cytokine-cytokine receptor interaction	5	4,7E-2	Ccl7, Cxcl12, Clcf1, Il18rap, Lif

Table S6a. Functional Annotation (UP_KEYWORDS) for all (213) MCAO DEGs

	Term	N	P-value	FDR	MCAO genes that did not respond to LPS	MCAO genes that responded to LPS
1	UP_KEYWORDS Secreted	38	3,0E-11	6,7E-9	20: Bpifb4, Ccl7, Timp1, Adm, Angpt2, Col11a2, Edn3, Eppin, Fgf1, Frzb, Hbegf, Inhba, Lif, Lox, Plaur, Prelp, Sfrp2, Serpina3n, Trh, Tsku	18: Adamts1, Ccl2, Cd14, Angptl4, Anxa1, Anxa2, B4galt1, Capg, Cp, Cyr61, Lgals3, Il11, Lbp, Mgp, Pla1a, Spp1, Serpine1, Serping1
2	UP_KEYWORDS Signal	68	3,3E-8	3,7E-6	40: Bpifb4, Ccl7, Cxcl12, Cd44, Fcrl2, Timp1, Acacb, Adm, Angpt2, Angptl2, Clcf1, Col11a2, C7, Emcn, Edn3, Eppin, Fbln2, Frzb, Glra1, Hbegf, Inhba, Itgax, Il18rap, Lif, Lox, Plaur, Pdpn, PVR, Prelp, Prom1,	28: Adamts1, Ccl2, Cd14, Fcgr2b, Tnfrsf12a, Tnfrsf1a, Angptl4, Cp, Csf2rb, Cyr61, Dhrs9, FAM187A, Fgl2, Gpnmb, Itgam, Il11, Lbp, Mgp,

					Ptgs2, Rxfp2, Rnf149, Ssc5d, Sfrp2, Serpina3n, Scube1, Trh, Tmem119, Tsku	Osmr, Ptx3, Pla1a, Ret, Spp1, Serpine1, Serping1, Thbs1, Tfpi2, Tnfrsf26
3	UP_KEYWORDS	Disulfide bond	50	9,9E-8	7,3E-6	27: Bpifb4, Ccl7, Cd44, Fcrl2, Timp1, Adm, Angpt2, Angptl2, C7, Edn3, Eppin, Fbln2, Frzb, Glra1, Hbegf, Inhba, Lif, Lox, Piezo1, Plaur, PVR, Prelp, Ptgs2, Rxfp2, Ssc5d, Sfrp2, Scube1	23: Adamts1, Ccl2, Cd14, Fcgr2b, S100a11, Tnfrsf1a, Angptl4, Anxa1, Cp, Clic1, Cyr61, FAM187A, Fgl2, Lgals3, Lbp, Msr1, Mgp, Niacr1, Osmr, Pla1a, P2ry12, Ret, Thbs1
4	UP_KEYWORDS	Glycoprotein	48	2,4E-7	1,3E-5	26: Abcc6, Bpifb4, Ccl7 , Cd44, Timp1, Angpt2, Creb3l1, Emcn, Ecel1, Emp1, Glra1, Hbegf, Hcn4, Inhba, Lif, Lox, Piezo1, Plaur, Pdpn, Kcnk13, Prelp, Ptgs2, Serpina3n, Slc38a5, Tmem204, Tsku	22: Adamts1, Ccl2, Cd14, Fcgr2b, Tnfrsf1a, A3galt2, Angptl4, Cp, Ch25h, FAM187A, Gpnmb, Lbp, Myc, Osmr, Pla1a, P2ry12, Ret, Spp1, Serpine1, Serping1, Slc44a4, Vim
5	UP_KEYWORDS	Innate immunity	8	2,6E-4	1,1E-2	0	8: Cd14, Fgr, Hck, Anxa1, Lgals3, Irf1, Lbp, Serping1
6	UP_KEYWORDS	Phosphoprotein	63	3,8E-4	1,4E-2	30: Pfkfb3, Abcc6, Cd44, Dbp, Fosl1, Pdlim1, Pdlim4, Timp1, Alox15, Emcn, Fgf1, Flnc, Gpd1, Gadd45a, Hmox1, Hcn4, L3mbtl1, March3, Ocln, Odc1, Plce1, Pygl, Piezo1, Pawr, Ppp1r15a, Rgs16, Serpina3n, Stat3, Sphk1, Tmem100	33: Fos, Fgr, Fcgr2b, Hck, Pim1, S100a11, Atf3, Anxa1, Anxa2, Aspg, Capg, Clic1, Cyr61, Des, Lgals3, Gfap, Gpnmb, Hspb1, Irf1, Mgp, Mt2A, Msn, Myc, Niacr1, Plek, P2ry12, Ret, Spp1, Slc16a3, Socs3, Tagln2, Vim, Zfp36
7	UP_KEYWORDS	Proto-oncogene	5	9,5E-4	3,0E-2	0	5: Fos, Fgr, Hck, Pim1, Myc
8	UP_KEYWORDS	Heparin-binding	5	1,7E-3	4,7E-2	3: Ccl7, Fgf1, Hbegf	

Table S6b. Functional Annotation (UP_KEYWORDS) for commonly responding to MCAO and LPS (84) MCAO DEGs

	Term	N	P-value	FDR	Genes
1	UP_KEYWORDS Innate immunity	8	5,8E-7	4,7E-5	Cd14, Fgr, Hck, Anxa1, Lgals3, Irf1, Lbp, Serping1
2	UP_KEYWORDS Secreted	18	6,4E-7	4,7E-5	Adamts1, Ccl2, Cd14, Angptl4, Anxa1, Anxa2, B4galt1, Capg, Cp, Cyr61, Lgals3, Il11, Lbp, Mgp, Pla1a, Spp1, Serpine1, Serping1
3	UP_KEYWORDS Proto-oncogene	5	2,6E-5	1,0E-3	Fos, Fgr, Hck, Pim1, Myc
4	UP_KEYWORDS Immunity	8	2,8E-5	1,0E-3	Cd14, Fgr, Hck, Anxa1, Lgals3, Irf1, Lbp, Serping1
5	UP_KEYWORDS Disulfide bond	23	3,6E-5	1,0E-3	Adamts1, Ccl2, Cd14, Fcgr2b, S100a11, Tnfrsf1a, Angptl4, Anxa1, Cp, Clic1, Cyr61, FAM187A, Fgl2, Lgals3, Lbp, Msr1, Mgp, Niacr1, Osmr, Pla1a, P2ry12, Ret, Thbs1
6	UP_KEYWORDS Phosphoprotein	33	4,2E-5	1,0E-3	Fos, Fgr, Fcgr2b, Hck, Pim1, S100a11, Atf3, Anxa1, Anxa2, Aspg, Capg, Clic1, Cyr61, Des, Lgals3, Gfap, Gpnmb, Hspb1, Irf1, Mgp, Mt2A, Msn, Myc, Niacr1, Plek, P2ry12, Ret, Spp1, Slc16a3, Socs3, Tagln2, Vim, Zfp36
7	UP_KEYWORDS Glycoprotein	22	6,7E-5	1,4E-3	Adamts1, Ccl2, Cd14, Fcgr2b, Tnfrsf1a, A3galt2, Angptl4, Cp, Ch25h, FAM187A, Gpnmb, Lbp, Myc, Osmr, Pla1a, P2ry12, Ret, Spp1, Serpine1, Serping1, Slc44a4, Vim
8	UP_KEYWORDS Signal	28	2,3E-4	4,3E-3	Adamts1, Ccl2, Cd14, Fcgr2b, Tnfrsf12a, Tnfrsf1a, Angptl4, Cp, Csf2rb, Cyr61, Dhrs9, FAM187A, Fgl2, Gpnmb, Itgam, Il11, Lbp, Mgp, Osmr, Ptx3, Pla1a, Ret, Spp1, Serpine1, Serping1, Thbs1, Tfpi2, Tnfrsf26
9	UP_KEYWORDS Ubl conjugation	11	1,2E-3	1,9E-2	Cebpd, Fos, Fgr, Hck, Pim1, Anxa1, Anxa2, Irf1, Myc, Vim, Zfp36

Table S6c Functional Annotation (UP_KEYWORDS) for not responding to LPS (129) MCAO DEGs

	Term		N	P-value	FDR	Genes
1	UP_KEYWORDS	Secreted	20	1,9E-5	3,2E-3	Bpifb4, Ccl7, Timp1, Adm, Angpt2, Col11a2, Edn3, Eppin, Fgf1, Frzb, Hbegf, Inhba, Lif, Lox, Plaur, Prelp, Sfrp2, Serpina3n, Trh, Tsku
2	UP_KEYWORDS	Signal	40	4,7E-5	4,0E-3	Bpifb4, Ccl7, Cxcl12, Cd44, Fcrl2, Timp1, Acacb, Adm, Angpt2, Angptl2, Clcf1, Col11a2, C7, Emcn, Edn3, Eppin, Fbln2, Frzb, Glra1, Hbegf, Inhba, Itgax, Il18rap, Lif, Lox, Plaur, Pdpn, PVR, Prelp, Prom1, Ptgs2, Rxfp2, Rnf149, Ssc5d, Sfrp2, Serpina3n, Scube1, Trh, Tmem119, Tsku
3	UP_KEYWORDS bond	Disulfide	27	7,1E-4	4,0E-2	Bpifb4, Ccl7, Cd44, Fcrl2, Timp1, Adm,Angpt2, Angptl2, C7, Edn3, Eppin, Fbln2, Frzb, Glra1, Hbegf, Inhba, Lif, Lox, Piezo1, Plaur, PVR, Prelp, Ptgs2, Rxfp2, Ssc5d, Sfrp2, Scube1
4	UP_KEYWORDS	Glycoprotein	26	9,8E-4	4,1E-2	Abcc6, Bpifb4, Ccl7 , Cd44, Timp1, Angpt2, Creb3l1, Emcn, Ecel1, Emp1, Glra1, Hbegf, Hcn4, Inhba, Lif, Lox, Piezo1, Plaur, Pdpn, Kcnk13, Prelp, Ptgs2, Serpina3n, Slc38a5, Tmem204, Tsku

Table S7a. Functional annotation clustering of all (213) MCAO DEGs

**Cluster 1** Enrichment Score: 2.58						
Category	Term	Count	P-value	FDR	MCAO genes that did not respond to LPS	MCAO genes that responded to LPS
UP_KEYWORDS	Innate immunity	8	2.4E-4	1.1E-2	0	Cd14, Fgr, Hck, Anxa1, Lgals3, Irf1, Lbp, Serping1
UP_KEYWORDS	Immunity	8	7.2E-3	1.4E-1	0	Cd14, Fgr, Hck, Anxa1, Lgals3, Irf1, Lbp, Serping1
GOTERM_BP_DIRECT	innate immune response	9	1.0E-2	2.3E-1	0	Clec5a, Cd14, Fgr, Hck, Anxa1, Lgals3, Lbp, Ptx3, Serping1

10 record(s)

ENSRNOG00000026306	C-type lectin domain family 5, member A(Clec5a)
ENSRNOG00000017819	CD14 molecule(Cd14)
ENSRNOG00000009912	FGR proto-oncogene, Src family tyrosine kinase(Fgr)
ENSRNOG00000009331	HCK proto-oncogene, Src family tyrosine kinase(Hck)
ENSRNOG00000017469	annexin A1(Anxa1)
ENSRNOG00000010645	galectin 3(Lgals3)
ENSRNOG00000008144	interferon regulatory factor 1(Irf1)
ENSRNOG00000014532	lipopolysaccharide binding protein(Lbp)
ENSRNOG00000012280	pentraxin 3(Ptx3)
ENSRNOG00000007457	serpin family G member 1(Serping1)

Table S7b. Functional annotation clustering of all up-regulated MCAO DEGs (182)

**Cluster 2** Enrichment Score: 2.99						
Category	Term	Count	P-value	FDR	MCAO genes that did not respond to LPS	MCAO genes that responded to LPS
UP_KEYWORDS	Innate immunity	8	9.1E-5	3.7E-3	0	Cd14, Fgr, Hck, Anxa1, Lgals3, Irf1, Lbp, Serping1
UP_KEYWORDS	Immunity	8	3.0E-3	6.8E-2	0	Cd14, Fgr, Hck, Anxa1, Lgals3, Irf1, Lbp,

						Serping1
GOTERM_BP_DIRECT	innate immune response	9	4.0E-3	1.1E-1	0	Clec5a, Cd14, Fgr, Hck, Anxa1, Lgals3, Lbp, Ptx3, Serping1

10 record(s)

ENSRNOG00000026306	C-type lectin domain family 5, member A(Clec5a)
ENSRNOG00000017819	CD14 molecule(Cd14)
ENSRNOG00000009912	FGR proto-oncogene, Src family tyrosine kinase(Fgr)
ENSRNOG00000009331	HCK proto-oncogene, Src family tyrosine kinase(Hck)
ENSRNOG00000017469	annexin A1(Anxa1)
ENSRNOG00000010645	galectin 3(Lgals3)
ENSRNOG00000008144	interferon regulatory factor 1(Irf1)
ENSRNOG00000014532	lipopolysaccharide binding protein(Lbp)
ENSRNOG00000012280	pentraxin 3(Ptx3)
ENSRNOG00000007457	serpin family G member 1(Serping1)
